# Hybrid metaheuristic optimized Catboost models for construction cost estimation of concrete solid slabs

**DOI:** 10.1038/s41598-025-06380-4

**Published:** 2025-07-01

**Authors:** Nanes Hassanin Elmasry, Mohamed Kamel Elshaarawy

**Affiliations:** 1 Civil Engineering Department, Faculty of Engineering, Tanta University, Tanta, 31733 Egypt; 2 Civil Engineering Department, Faculty of Engineering, Horus University-Egypt, New Damietta, 34517 Egypt

**Keywords:** Construction cost prediction, CatBoost, Concrete, Solid slabs, Metaheuristic optimization, SHAP analysis, Engineering, Civil engineering

## Abstract

Accurate construction cost prediction is essential for effective project planning and resource allocation, particularly in the competitive construction industry. This study introduces an advanced approach to predicting the costs of concrete solid slabs by combining the Categorical Boosting (CatBoost) model with three hybrid metaheuristic optimization models: Phasor Particle Swarm Optimization (PPSO-CatBoost), Dwarf Mongoose Optimization (DMO-CatBoost), and Atom Search Optimization (ASO-CatBoost). These hybrid models were designed to optimize critical hyperparameters, including depth, learning rate, and iterations, and were benchmarked against a standalone CatBoost model using various performance metrics, such as residual error cumulative distribution (REC) curves, scatter plots, violin plots, and quantitative measures. The results reveal that the hybrid models consistently outperform the standalone CatBoost model, with ASO-CatBoost achieving the best overall performance with determination coefficient (*R*^2^) of 0.981 and root-mean-squared-error (RMSE) of 1.222 $/m^2^. The ASO-CatBoost exhibited superior accuracy and generalization, characterized by minimal residual errors and close alignment with actual cost values during both training and testing phases. SHapley Additive exPlanations (SHAP) analysis identified the Tributary Area ($/m^2^) as the most influential variable, followed by Concrete ($/m^3^), underscoring the importance of these inputs in cost prediction. Additionally, a Python-based graphical user interface (GUI) was developed, enabling practical and user-friendly cost estimation in real-world applications.

## Introduction

The construction industry generates vast amounts of data during different phases of a project, but much of this data remains untapped^1–3^. By leveraging machine learning (ML), a branch of artificial intelligence (AI), it becomes possible to process this complex information and derive meaningful patterns that can address critical challenges in construction management^4–7^. To achieve this, it is essential to develop frameworks that deliver precise predictive models, quantify uncertainties in forecasts, and provide insights into the importance of input factors^8^. In construction, selecting the right combination of designs, materials, and methods is essential for meeting project goals.

Value engineering (VE) provides a systematic methodology for optimizing these choices by identifying cost-effective alternatives that meet technical requirements and maintain performance standards^9^. VE aims to maximize the value of investments by evaluating various design options for both immediate and long-term benefits^10,11^. For instance, structural systems for medium- and high-rise buildings often involve choices between concrete systems (e.g., one-way slabs, two-way slabs, waffle slabs) and steel systems (e.g., beams, girders, joists). However, the number of alternatives considered is often restricted due to time and resource constraints, emphasizing the need for accurate early-stage cost predictions^12,13^.

Traditional cost estimation approaches, such as detailed quantity take-offs, are time-intensive and heavily reliant on complete project documentation. Simplified methods, like comparative cost estimation, often assume linear relationships between costs and design variables, leading to inaccuracies^14^. To overcome these limitations, researchers have explored advanced computational tools, including machine learning and simulation techniques, to streamline the cost prediction process. These innovations are essential in improving efficiency and ensuring more reliable outcomes in construction projects. Mantel et al.^15^ identified three critical criteria for measuring the performance of construction projects: completion on or ahead of schedule, adherence to the estimated budget, and meeting client expectations. These criteria, collectively referred to as performance targets, are fundamental benchmarks for project success. When any of these criteria are unmet, the project management process is considered unsuccessful. Cost and time management are crucial components of construction project planning, as delays and budget overruns are common challenges caused by various factors.

For instance, a survey by Akalya et al.^16^ revealed that time overruns are often driven by high-quality demands, fluctuating market rates for construction materials, and contract modifications. Similarly, the main causes of cost overruns include changes in material prices, high transportation costs, and alterations in material specifications. Other studies have highlighted additional factors contributing to these issues. Ali and Kamaruzzaman^17^ pointed to inaccurate original cost estimations, while Subramani et al.^18^ emphasized inadequate contract and schedule management, delays in providing designs, and inefficiencies in correcting errors during construction. Memon et al.^19^ identified poor site management, financial difficulties, cash flow issues, and insufficient contractor experience as key contributors to cost overruns.

The construction industry has also faced increased competition due to slow global economic growth, rising material costs, inflation, and deflated credit policies^20^. These challenges amplify the need for effective cost management strategies. Additionally, clients consistently demand high-quality performance at reduced costs, underscoring the importance of cost control measures and techniques for cost reduction^21^. Since materials and equipment account for approximately 70% of total project costs, effective control over these components can significantly reduce overall expenses^19^.

Recent studies have shown that artificial neural networks (ANNs) can generate reliable cost estimates using historical data^22,23^. Elfahham^24^ developed a formula for calculating the construction cost index (CCI) for concrete structures, demonstrating that autoregressive time series models outperformed ANN and regression models in predictive accuracy. Similarly, El-Kholy et al.^25^ evaluated ANN and regression models for estimating cost contingencies due to fluctuations in steel prices, recommending regression-based models for their practicality and precision. Alshamrani^26^ developed a regression model for mid-rise green office buildings, which enables stakeholders to compare the costs of green and traditional designs. Deepa et al.^27^ studied the application of a hybrid machine learning approach for early cost estimation of pile foundations, addressing the persistent challenge of cost overruns in construction projects. They highlighted that conventional cost estimation methods, including manual computations, are often time-consuming, error-prone, and inefficient. To enhance accuracy and efficiency, the study integrates ML techniques with ANNs, leveraging their combined predictive capabilities. By utilizing cost-related data from five different construction projects in India, the proposed model demonstrated a 97.42% accuracy in predicting pile foundation costs. Cheng et al.^28^ introduced a hybrid model combining genetic algorithms, fuzzy logic, and neural networks for conceptual cost estimation. Das et al.^29^ applied natural gradient boosting (NGBoost) to predict construction costs, achieving high accuracy with *R*^1^ values of 0.992 for training and 0.985 for testing. Sensitivity analysis identified formwork as the most influential factor, contributing around 41% to overall costs.

### Research gap

In this context, this research investigates the forecasting of the construction cost of concrete slabs using CatBoost models optimized with metaheuristic algorithms. The reason for selecting the CatBoost model was due to its effectiveness use in various fields where it has often been used to overcome the challenges associated with high accuracy in supervised learning^30,31^. Nevertheless, comprehensive research assessing the complex issues of forecasting the construction cost using the CatBoost model is limited. Thus, hybrid CatBoost models were constructed in this study using newly emerging and competitive optimization techniques to extend the limits of CatBoost and find the best combinations of hyperparameters. Finally, a flexible and novel GUI has been developed based on the best predicting model in this work. This GUI allows users to easily and effectively use the model developed to predict the construction cost of concrete slabs. Thus, it provides a user-friendly tool for engineers and researchers to visualize results and make predictions in various scenarios to optimize design processes and facilitate decision-making^32^.

## Materials and methods

**Fig. 1:**
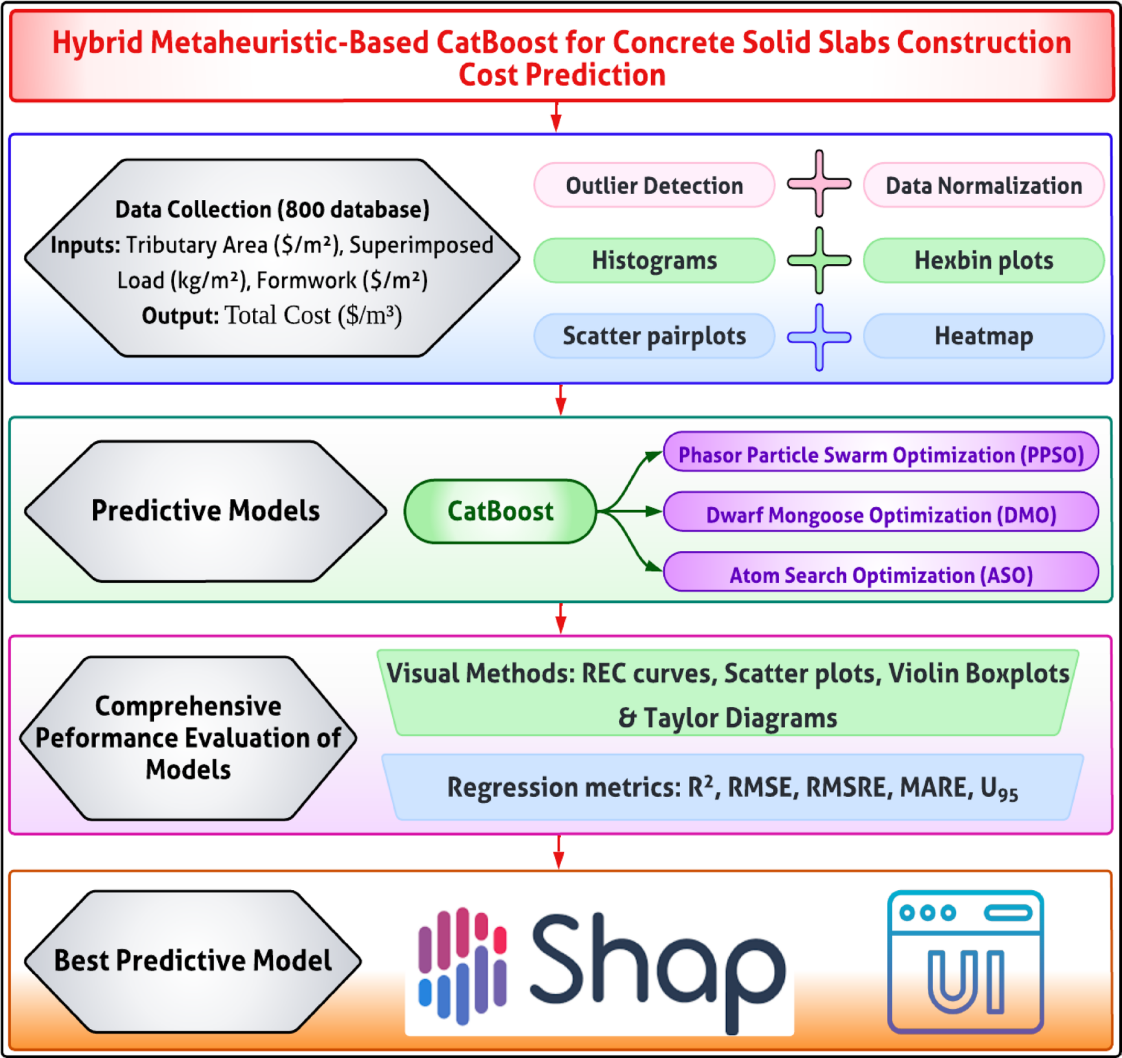
Methodological flowchart adopted in this study.

Figure 1 illustrates the methodological framework adopted for predicting the construction costs of concrete slabs using a hybrid machine learning approach. The process begins with data collection and preprocessing, which includes statistical analysis, correlation mapping, and sensitivity evaluation to ensure data quality and relevance. The CatBoost algorithm serves as the core predictive model, with its hyperparameters optimized using three advanced metaheuristic techniques: PPSO, DMO, and ASO. The dataset is divided into training and testing sets to validate model performance. Evaluation is conducted through a combination of visual tools, such as regression error curves and scatter plots and statistical metrics to ensure robust assessment. The methodology concludes with explainability analysis using SHAP to interpret feature contributions and the development of an interactive GUI, facilitating practical application and user engagement. This systematic approach ensures accuracy, interpretability, and real-world usability.

### Database collection

The basis of ML methodology is a data set; therefore, the collection of a comprehensive and precise data set is a fundamental step in the development of ML prediction models^33,34^. Overall, a database with a sample size exceeding 10–20 times the number of independent parameters used to construct regression models is critical for building reliable and highly generalizable models^35–41^. The size, diversity, and quality of the data set are among the key factors affecting the accuracy and performance of ML algorithms. So, the development of an effective ML prediction model includes a meticulous data collection process and a disciplined approach in the analysis phases. This database was used to develop the predictive models in this study. Table 1 lists the collected dataset to ensure reproducibility and practical applicability.

The collected dataset was derived from RSMeans Assemblies and Unit Cost Data Books^42^covering the period from 1998 to 2018. The dataset includes a total of 800 data instances related to structural floor assemblies, specifically one-way solid concrete slabs used in medium- and high-rise buildings. The dependent variable is the total construction cost of the slab system, expressed in dollars per square meter ($/m^2^). The input (independent) variables comprise tributary area (m^2^), superimposed load (kg/m^2^), unit cost of formwork ($/m^2^), and unit cost of concrete ($/m^3^). The year of estimation was not considered an input variable, as the use of multi-year data was solely intended to capture variations in component unit costs across time. RSMeans was selected as the data source due to its comprehensive, annually updated database that reflects current construction material, labor, and equipment costs. It also incorporates new construction methodologies, productivity rates, and localized market conditions to ensure reliability. Independent variables were obtained from the RSMeans Unit Cost Data Book^42^while the dependent variable was sourced from the RSMeans Assemblies Data Book. This dataset served as the foundation for model development, training, validation, and comparative performance evaluation.

**Table 1:** Summary of the collected dataset of the concrete solid slabs^43^.

#	Inputs				Output
	Tributary Area ($/m^2^)	Superimposed Load (kg/m^2^)	Formwork ($/m^2^)	Concrete ($/m^3^)	Total Cost ($/m^2^)
1	20.90	195.28	46.98	83.09	82.8828
2	20.90	366.15	46.98	83.09	83.0981
3	20.90	610.25	46.98	83.09	84.7127
4	20.90	195.28	49.15	79.25	85.0356
5	20.90	366.15	49.15	79.25	85.2509
6	20.90	976.40	46.98	83.09	85.3585
7	20.90	610.25	49.15	79.25	86.6502
8	20.90	976.40	49.15	79.25	87.2960
9	37.16	195.28	46.98	83.09	87.8342
10	27.87	195.28	46.98	83.09	87.9419
11	20.90	195.28	50.95	79.17	88.2648
12	20.90	195.28	51.60	83.30	88.3724
13	20.90	195.28	49.91	90.79	88.4801
14	20.90	366.15	49.91	90.79	88.6954
15	20.90	366.15	50.95	79.17	88.9106
16	37.16	195.28	49.15	79.25	89.4488
17	20.90	366.15	51.60	83.30	89.5565
18	27.87	195.28	49.15	79.25	89.9870
19	20.90	610.25	49.91	90.79	90.2023
20	20.90	195.28	51.00	93.10	90.4176
21	20.90	366.15	51.00	93.10	90.6329
22	27.87	195.28	50.95	79.17	90.6329
23	20.90	976.40	49.91	90.79	90.9558
24	27.87	366.15	46.98	83.09	91.0634
25	37.16	366.15	46.98	83.09	91.2787
26	20.90	610.25	50.95	79.17	91.3864
27	27.87	195.28	51.60	83.30	91.3864
28	20.90	610.25	51.60	83.30	92.0322
29	20.90	610.25	51.00	93.10	92.1398
30	27.87	366.15	49.15	79.25	93.0010
31	37.16	195.28	50.95	79.17	93.0010
32	37.16	366.15	49.15	79.25	93.2162
33	27.87	366.15	50.95	79.17	93.2162
34	20.90	976.40	51.00	93.10	93.4315
35	37.16	195.28	49.91	90.79	93.6468
36	27.87	195.28	49.91	90.79	93.7544
37	27.87	366.15	51.60	83.30	93.8621
38	37.16	195.28	51.60	83.30	94.2926
39	20.90	976.40	50.95	79.17	94.6156
40	20.90	195.28	55.50	90.18	94.9385
41	20.90	195.28	54.04	99.25	95.0461
42	46.45	195.28	46.98	83.09	95.1538
43	20.90	366.15	54.04	99.25	95.1538
44	37.16	195.28	51.00	93.10	95.2614
45	20.90	976.40	51.60	83.30	95.2614
46	37.16	610.25	46.98	83.09	95.3690
47	27.87	195.28	51.00	93.10	95.3690
48	27.87	610.25	46.98	83.09	95.9072
49	27.87	976.40	46.98	83.09	96.1225
50	20.90	366.15	55.50	90.18	96.2302
51	37.16	976.40	46.98	83.09	96.3378
52	37.16	366.15	50.95	79.17	96.4454
53	20.90	610.25	54.04	99.25	96.7684
54	58.06	195.28	46.98	83.09	96.8760
55	27.87	610.25	50.95	79.17	96.8760
56	46.45	366.15	46.98	83.09	96.9836
57	27.87	366.15	49.91	90.79	96.9836
58	20.90	195.28	47.08	83.24	97.0913
59	20.90	195.28	56.69	92.93	97.0913
60	46.45	195.28	49.15	79.25	97.1989
61	37.16	366.15	51.60	83.30	97.1989
62	27.87	610.25	49.15	79.25	97.3066
63	37.16	610.25	49.15	79.25	97.3066
64	37.16	366.15	49.91	90.79	97.3066
65	27.87	610.25	51.60	83.30	97.6295
66	20.90	976.40	54.04	99.25	97.9524
67	27.87	976.40	49.15	79.25	98.1677
68	37.16	976.40	49.15	79.25	98.1677
69	27.87	195.28	55.50	90.18	98.1677
70	20.90	366.15	56.69	92.93	98.3830
71	20.90	610.25	55.50	90.18	98.7059
72	20.90	366.15	47.08	83.24	98.8135
73	46.45	366.15	49.15	79.25	98.8135
74	58.06	195.28	49.15	79.25	98.8135
75	27.87	976.40	50.95	79.17	98.9212
76	46.45	195.28	50.95	79.17	99.0288
77	27.87	366.15	51.00	93.10	99.1364
78	20.90	195.28	55.77	103.87	99.2441
79	37.16	366.15	51.00	93.10	99.4594
80	20.90	366.15	55.77	103.87	99.5670
81	27.87	976.40	51.60	83.30	99.5670
82	20.90	195.28	57.45	99.13	99.5670
83	27.87	195.28	56.69	92.93	99.8899
84	37.16	195.28	54.04	99.25	100.1052
85	27.87	195.28	47.08	83.24	100.4281
86	46.45	195.28	51.60	83.30	100.4281
87	20.90	195.28	55.90	87.17	100.4281
88	20.90	195.28	49.24	79.20	100.5358
89	58.06	195.28	55.90	87.17	100.5358
90	27.87	195.28	54.04	99.25	100.6434
91	58.06	195.28	56.02	80.81	100.7510
92	58.06	366.15	46.98	83.09	100.8587
93	20.90	610.25	56.69	92.93	100.9663
94	27.87	195.28	55.90	87.17	100.9663
95	37.16	195.28	55.50	90.18	101.0740
96	20.90	195.28	56.02	80.81	101.0740
97	20.90	366.15	55.90	87.17	101.0740
98	20.90	610.25	55.77	103.87	101.2892
99	27.87	366.15	55.50	90.18	101.2892
100	37.16	195.28	55.90	87.17	101.2892
101	20.90	366.15	57.45	99.13	101.3969
102	20.90	366.15	56.02	80.81	101.7198
103	37.16	195.28	56.02	80.81	101.7198
104	20.90	366.15	49.24	79.20	101.8274
105	58.06	195.28	50.95	79.17	101.8274
106	46.45	195.28	49.91	90.79	101.9351
107	20.90	976.40	55.50	90.18	102.0427
108	27.87	195.28	56.02	80.81	102.0427
109	27.87	610.25	49.91	90.79	102.1504
110	37.16	610.25	49.91	90.79	102.1504
111	46.45	610.25	46.98	83.09	102.2580
112	46.45	195.28	55.90	87.17	102.2580
113	27.87	976.40	49.91	90.79	102.3656
114	20.90	976.40	55.77	103.87	102.3656
115	20.90	610.25	47.08	83.24	102.4733
116	37.16	610.25	50.95	79.17	102.4733
117	20.90	610.25	55.90	87.17	102.4733
118	27.87	195.28	45.15	77.58	102.6886
119	58.06	195.28	51.60	83.30	102.6886
120	27.87	195.28	57.45	99.13	102.7962
121	46.45	195.28	56.02	80.81	102.7962
122	27.87	366.15	56.69	92.93	102.9038
123	20.90	195.28	45.15	77.58	103.0115
124	20.90	195.28	58.43	103.95	103.0115
125	27.87	195.28	49.24	79.20	103.1191
126	37.16	976.40	49.91	90.79	103.1191
127	20.90	610.25	56.02	80.81	103.1191
128	58.06	366.15	49.15	79.25	103.2268
129	37.16	610.25	51.60	83.30	103.3344
130	37.16	195.28	56.69	92.93	103.3344
131	27.87	366.15	55.90	87.17	103.4420
132	20.90	366.15	45.15	77.58	103.5497
133	58.06	195.28	49.91	90.79	103.7650
134	46.45	610.25	49.15	79.25	103.8726
135	46.45	366.15	49.91	90.79	103.8726
136	37.16	195.28	45.15	77.58	103.9802
137	20.90	610.25	57.45	99.13	103.9802
138	27.87	366.15	56.02	80.81	103.9802
139	27.87	366.15	54.04	99.25	104.0879
140	46.45	195.28	51.00	93.10	104.1955
141	37.16	366.15	54.04	99.25	104.3032
142	37.16	976.40	50.95	79.17	104.3032
143	20.90	976.40	56.69	92.93	104.3032
144	27.87	610.25	51.00	93.10	104.4108
145	37.16	610.25	51.00	93.10	104.5184
146	27.87	976.40	51.00	93.10	104.6261
147	27.87	195.28	55.77	103.87	104.7337
148	37.16	195.28	55.77	103.87	104.7337
149	37.16	366.15	55.50	90.18	104.7337
150	46.45	976.40	46.98	83.09	104.8414
151	20.90	366.15	58.43	103.95	104.8414
152	46.45	366.15	50.95	79.17	104.9490
153	58.06	610.25	46.98	83.09	105.0566
154	37.16	976.40	51.60	83.30	105.0566
155	27.87	610.25	55.50	90.18	105.0566
156	46.45	195.28	45.15	77.58	105.1643
157	37.16	195.28	47.08	83.24	105.1643
158	20.90	610.25	45.15	77.58	105.2719
159	46.45	366.15	51.60	83.30	105.2719
160	58.06	195.28	45.15	77.58	105.3796
161	37.16	976.40	51.00	93.10	105.3796
162	20.90	195.28	51.05	92.93	105.4872
163	27.87	195.28	47.20	74.35	105.7025
164	69.68	195.28	55.90	87.17	105.7025
165	37.16	195.28	57.45	99.13	105.9178
166	27.87	366.15	45.15	77.58	106.0254
167	20.90	610.25	49.24	79.20	106.0254
168	58.06	195.28	51.00	93.10	106.0254
169	69.68	195.28	56.02	80.81	106.0254
170	46.45	366.15	51.00	93.10	106.1330
171	58.06	366.15	50.95	79.17	106.1330
172	27.87	366.15	57.45	99.13	106.1330
173	20.90	195.28	47.20	74.35	106.2407
174	37.16	195.28	47.20	74.35	106.3483
175	20.90	976.40	55.90	87.17	106.4560
176	46.45	976.40	49.15	79.25	106.5636
177	27.87	976.40	55.50	90.18	106.5636
178	58.06	366.15	55.90	87.17	106.6712
179	58.06	610.25	49.15	79.25	106.7789
180	27.87	610.25	56.69	92.93	106.7789
181	37.16	366.15	55.90	87.17	106.7789
182	37.16	366.15	56.69	92.93	106.9942
183	58.06	366.15	56.02	80.81	106.9942
184	58.06	366.15	51.60	83.30	107.1018
185	27.87	195.28	58.43	103.95	107.1018
186	20.90	366.15	47.20	74.35	107.2094
187	69.68	195.28	50.95	79.17	107.2094
188	20.90	976.40	56.02	80.81	107.2094
189	20.90	976.40	57.45	99.13	107.3171
190	37.16	366.15	56.02	80.81	107.3171
191	20.90	366.15	51.05	92.93	107.5324
192	46.45	195.28	55.50	90.18	107.5324
193	20.90	610.25	58.43	103.95	107.5324
194	46.45	366.15	55.90	87.17	107.5324
195	37.16	195.28	49.24	79.20	107.6400
196	58.06	195.28	59.59	96.08	107.6400
197	69.68	195.28	45.15	77.58	107.7476
198	46.45	195.28	47.20	74.35	107.9629
199	46.45	366.15	56.02	80.81	108.1782
200	37.16	610.25	55.90	87.17	108.1782
201	83.61	195.28	55.90	87.17	108.2858
202	37.16	195.28	59.59	96.08	108.2858
203	20.90	610.25	47.20	74.35	108.3935
204	27.87	366.15	47.08	83.24	108.3935
205	58.06	366.15	49.91	90.79	108.3935
206	83.61	195.28	56.02	80.81	108.3935
207	58.06	195.28	47.20	74.35	108.5011
208	20.90	195.28	59.59	96.08	108.5011
209	27.87	366.15	55.77	103.87	108.6088
210	69.68	195.28	51.60	83.30	108.7164
211	46.45	976.40	88.13	174.17	108.7164
212	20.90	366.15	59.59	96.08	108.7164
213	20.90	195.28	47.92	84.85	108.9317
214	20.90	195.28	49.97	90.51	108.9317
215	27.87	195.28	47.92	84.85	109.0393
216	27.87	195.28	49.97	90.51	109.0393
217	37.16	366.15	55.77	103.87	109.0393
218	46.45	195.28	54.04	99.25	109.1470
219	27.87	610.25	55.90	87.17	109.1470
220	27.87	195.28	59.59	96.08	109.1470
221	69.68	366.15	55.90	87.17	109.2546
222	46.45	195.28	59.59	96.08	109.2546
223	27.87	610.25	54.04	99.25	109.3622
224	37.16	610.25	54.04	99.25	109.3622
225	27.87	976.40	56.69	92.93	109.3622
226	37.16	610.25	56.02	80.81	109.3622
227	27.87	195.28	51.05	92.93	109.4699
228	27.87	610.25	57.45	99.13	109.4699
229	27.87	976.40	54.04	99.25	109.5775
230	37.16	366.15	57.45	99.13	109.5775
231	27.87	366.15	47.20	74.35	109.6852
232	20.90	976.40	45.15	77.58	109.6852
233	46.45	610.25	49.91	90.79	109.6852
234	37.16	195.28	58.43	103.95	109.6852
235	69.68	366.15	56.02	80.81	109.6852
236	46.45	195.28	56.69	92.93	109.9004
237	27.87	366.15	58.43	103.95	109.9004
238	27.87	610.25	56.02	80.81	109.9004
239	58.06	195.28	60.78	98.63	109.9004
240	37.16	195.28	60.17	87.01	110.0081
241	20.90	366.15	47.92	84.85	110.1157
242	20.90	366.15	49.97	90.51	110.1157
243	20.90	610.25	59.59	96.08	110.1157
244	20.90	195.28	60.78	98.63	110.1157
245	27.87	610.25	45.15	77.58	110.2234
246	58.06	366.15	51.00	93.10	110.2234
247	37.16	976.40	54.04	99.25	110.2234
248	69.68	195.28	47.20	74.35	110.3310
249	20.90	976.40	47.08	83.24	110.4386
250	46.45	610.25	50.95	79.17	110.4386
251	37.16	195.28	60.78	98.63	110.4386
252	58.06	195.28	55.50	90.18	110.5463
253	37.16	195.28	47.92	84.85	110.7616
254	37.16	195.28	49.97	90.51	110.7616
255	27.87	195.28	60.78	98.63	110.7616
256	46.45	195.28	47.08	83.24	110.8692
257	20.90	366.15	60.78	98.63	110.8692
258	37.16	610.25	55.50	90.18	111.0845
259	20.90	195.28	54.06	99.40	111.1921
260	46.45	610.25	51.60	83.30	111.4074
261	20.90	976.40	58.43	103.95	111.4074
262	27.87	366.15	49.24	79.20	111.5150
263	46.45	610.25	51.00	93.10	111.5150
264	27.87	366.15	59.59	96.08	111.5150
265	46.45	366.15	54.04	99.25	111.6227
266	58.06	610.25	50.95	79.17	111.6227
267	46.45	195.28	60.17	87.01	111.6227
268	20.90	610.25	51.05	92.93	111.7303
269	58.06	195.28	54.04	99.25	111.7303
270	20.90	195.28	63.26	116.18	111.7303
271	20.90	610.25	47.92	84.85	111.8380
272	27.87	195.28	49.00	87.28	111.8380
273	20.90	610.25	49.97	90.51	111.8380
274	83.61	366.15	55.90	87.17	111.8380
275	46.45	976.40	49.91	90.79	111.9456
276	27.87	976.40	57.45	99.13	111.9456
277	37.16	366.15	111.62	146.38	111.9456
278	37.16	366.15	45.15	77.58	112.0532
279	20.90	195.28	49.00	87.28	112.0532
280	20.90	366.15	63.26	116.18	112.0532
281	58.06	610.25	51.60	83.30	112.0532
282	46.45	195.28	60.78	98.63	112.0532
283	37.16	366.15	60.17	87.01	112.0532
284	46.45	195.28	47.92	84.85	112.1609
285	46.45	195.28	49.97	90.51	112.1609
286	58.06	610.25	49.91	90.79	112.1609
287	83.61	195.28	50.95	79.17	112.1609
288	27.87	976.40	55.90	87.17	112.1609
289	27.87	976.40	56.02	80.81	112.2685
290	20.90	610.25	60.78	98.63	112.2685
291	58.06	195.28	47.92	84.85	112.4838
292	58.06	195.28	49.97	90.51	112.4838
293	20.90	366.15	49.00	87.28	112.6991
294	83.61	366.15	56.02	80.81	112.6991
295	97.55	195.28	55.90	87.17	112.6991
296	69.68	366.15	50.95	79.17	112.8067
297	37.16	976.40	55.50	90.18	112.8067
298	20.90	976.40	47.20	74.35	112.9144
299	37.16	195.28	49.00	87.28	112.9144
300	58.06	195.28	56.69	92.93	112.9144
301	97.55	195.28	56.02	80.81	112.9144
302	69.68	195.28	59.59	96.08	112.9144
303	20.90	366.15	54.06	99.40	113.0220
304	27.87	366.15	47.92	84.85	113.1296
305	27.87	366.15	49.97	90.51	113.1296
306	46.45	366.15	55.50	90.18	113.1296
307	46.45	195.28	57.45	99.13	113.2373
308	27.87	366.15	60.78	98.63	113.2373
309	20.90	976.40	49.24	79.20	113.4526
310	37.16	610.25	56.69	92.93	113.4526
311	37.16	976.40	55.90	87.17	113.4526
312	113.81	195.28	55.90	87.17	113.4526
313	58.06	195.28	47.08	83.24	113.5602
314	46.45	610.25	55.90	87.17	113.5602
315	37.16	610.25	60.17	87.01	113.5602
316	113.81	195.28	56.02	80.81	113.6678
317	97.55	366.15	55.90	87.17	113.6678
318	27.87	610.25	58.43	103.95	113.7755
319	58.06	366.15	45.15	77.58	113.8831
320	83.61	195.28	51.60	83.30	113.8831
321	37.16	366.15	58.43	103.95	113.8831
322	46.45	366.15	60.17	87.01	113.8831
323	46.45	195.28	49.24	79.20	113.9908
324	46.45	976.40	50.95	79.17	113.9908
325	58.06	366.15	59.59	96.08	113.9908
326	58.06	195.28	60.17	87.01	113.9908
327	27.87	610.25	47.20	74.35	114.0984
328	46.45	195.28	55.77	103.87	114.0984
329	20.90	610.25	63.26	116.18	114.0984
330	46.45	610.25	56.02	80.81	114.0984
331	37.16	366.15	47.08	83.24	114.2060
332	46.45	976.40	51.00	93.10	114.2060
333	37.16	976.40	56.02	80.81	114.2060
334	20.90	610.25	49.00	87.28	114.3137
335	46.45	195.28	49.00	87.28	114.3137
336	69.68	366.15	51.60	83.30	114.3137
337	37.16	610.25	55.77	103.87	114.4213
338	20.90	976.40	59.59	96.08	114.4213
339	20.90	195.28	62.21	106.26	114.4213
340	97.55	366.15	56.02	80.81	114.5290
341	37.16	366.15	59.59	96.08	114.5290
342	69.68	195.28	47.92	84.85	114.6366
343	69.68	195.28	49.97	90.51	114.6366
344	27.87	195.28	54.06	99.40	114.6366
345	46.45	366.15	59.59	96.08	114.6366
346	37.16	610.25	111.62	146.38	114.6366
347	58.06	195.28	62.21	106.26	114.7442
348	46.45	366.15	45.15	77.58	114.8519
349	69.68	366.15	45.15	77.58	114.8519
350	37.16	195.28	51.05	92.93	114.8519
351	27.87	610.25	55.77	103.87	114.8519
352	46.45	976.40	51.60	83.30	114.8519
353	58.06	366.15	55.50	90.18	114.8519
354	37.16	195.28	62.21	106.26	114.8519
355	37.16	366.15	47.20	74.35	114.9595
356	58.06	610.25	51.00	93.10	114.9595
357	58.06	195.28	49.00	87.28	115.0672
358	27.87	976.40	55.77	103.87	115.1748
359	37.16	976.40	55.77	103.87	115.1748
360	37.16	976.40	56.69	92.93	115.1748
361	20.90	366.15	62.21	106.26	115.1748
362	58.06	976.40	50.95	79.17	115.3901
363	46.45	366.15	56.69	92.93	115.4977
364	27.87	195.28	62.21	106.26	115.4977
365	20.90	976.40	63.26	116.18	115.6054
366	69.68	195.28	60.17	87.01	115.6054
367	58.06	366.15	54.04	99.25	115.7130
368	20.90	195.28	66.45	116.34	115.7130
369	69.68	195.28	60.78	98.63	115.7130
370	27.87	366.15	49.00	87.28	115.8206
371	46.45	195.28	62.21	106.26	115.9283
372	58.06	195.28	49.24	79.20	116.0359
373	37.16	610.25	59.59	96.08	116.0359
374	46.45	610.25	60.17	87.01	116.0359
375	58.06	195.28	57.45	99.13	116.2512
376	27.87	976.40	58.43	103.95	116.2512
377	27.87	610.25	59.59	96.08	116.2512
378	83.61	195.28	59.59	96.08	116.2512
379	20.90	976.40	60.78	98.63	116.2512
380	20.90	976.40	47.92	84.85	116.3588
381	20.90	976.40	49.97	90.51	116.3588
382	69.68	610.25	50.95	79.17	116.4665
383	58.06	976.40	51.60	83.30	116.4665
384	37.16	366.15	49.24	79.20	116.5741
385	20.90	195.28	55.87	104.25	116.5741
386	20.90	610.25	62.21	106.26	116.5741
387	58.06	195.28	55.77	103.87	116.6818
388	37.16	610.25	57.45	99.13	116.6818
389	69.68	195.28	55.50	90.18	116.7894
390	58.06	366.15	56.69	92.93	116.7894
391	37.16	366.15	60.78	98.63	116.7894
392	58.06	366.15	60.78	98.63	116.7894
393	27.87	610.25	47.08	83.24	116.8970
394	20.90	195.28	66.19	124.64	116.8970
395	46.45	610.25	54.04	99.25	117.0047
396	69.68	610.25	55.90	87.17	117.0047
397	58.06	366.15	47.20	74.35	117.1123
398	37.16	610.25	45.15	77.58	117.1123
399	46.45	195.28	58.43	103.95	117.1123
400	20.90	366.15	66.19	124.64	117.2200
401	69.68	366.15	59.59	96.08	117.2200
402	58.06	366.15	60.17	87.01	117.2200
403	20.90	610.25	54.06	99.40	117.3276
404	46.45	366.15	55.77	103.87	117.3276
405	69.68	195.28	49.00	87.28	117.4352
406	130.06	195.28	56.02	80.81	117.4352
407	27.87	610.25	47.92	84.85	117.5429
408	27.87	195.28	51.89	92.93	117.5429
409	27.87	610.25	49.97	90.51	117.5429
410	69.68	366.15	47.20	74.35	117.6505
411	130.06	195.28	55.90	87.17	117.6505
412	46.45	366.15	60.78	98.63	117.6505
413	69.68	195.28	47.08	83.24	117.7582
414	20.90	366.15	66.45	116.34	117.7582
415	37.16	195.28	63.40	96.22	117.7582
416	46.45	366.15	47.20	74.35	117.8658
417	27.87	366.15	51.05	92.93	117.8658
418	83.61	366.15	50.95	79.17	117.8658
419	69.68	610.25	51.60	83.30	117.9734
420	37.16	976.40	57.45	99.13	117.9734
421	37.16	195.28	68.89	80.35	117.9734
422	27.87	366.15	62.21	106.26	118.0811
423	69.68	610.25	56.02	80.81	118.1887
424	37.16	610.25	47.08	83.24	118.2964
425	83.61	195.28	45.15	77.58	118.4040
426	20.90	366.15	51.89	92.93	118.4040
427	20.90	366.15	55.87	104.25	118.4040
428	27.87	195.28	63.26	116.18	118.4040
429	58.06	610.25	55.90	87.17	118.4040
430	37.16	195.28	51.89	92.93	118.5116
431	37.16	195.28	63.26	116.18	118.5116
432	27.87	610.25	60.78	98.63	118.5116
433	37.16	366.15	47.92	84.85	118.7269
434	37.16	366.15	49.97	90.51	118.7269
435	83.61	610.25	55.90	87.17	118.7269
436	69.68	366.15	60.17	87.01	118.7269
437	58.06	610.25	56.02	80.81	118.8346
438	113.81	366.15	56.02	80.81	118.8346
439	37.16	610.25	60.78	98.63	118.8346
440	46.45	366.15	57.45	99.13	118.9422
441	27.87	976.40	45.15	77.58	119.0498
442	20.90	976.40	49.00	87.28	119.0498
443	83.61	610.25	56.02	80.81	119.0498
444	37.16	976.40	60.17	87.01	119.0498
445	46.45	976.40	55.90	87.17	119.1575
446	83.61	195.28	60.78	98.63	119.1575
447	20.90	976.40	51.05	92.93	119.2651
448	69.68	195.28	56.69	92.93	119.2651
449	97.55	610.25	55.90	87.17	119.2651
450	113.81	366.15	55.90	87.17	119.2651
451	113.81	610.25	56.02	80.81	119.3728
452	46.45	976.40	54.04	99.25	119.4804
453	97.55	195.28	50.95	79.17	119.4804
454	113.81	610.25	55.90	87.17	119.4804
455	46.45	195.28	68.89	80.35	119.4804
456	83.61	366.15	51.60	83.30	119.5880
457	27.87	610.25	49.00	87.28	119.6957
458	27.87	976.40	59.59	96.08	119.6957
459	37.16	366.15	63.40	96.22	119.6957
460	27.87	610.25	49.24	79.20	119.8033
461	46.45	195.28	63.40	96.22	119.8033
462	46.45	610.25	55.50	90.18	119.9110
463	37.16	366.15	68.89	80.35	119.9110
464	20.90	610.25	51.89	92.93	120.0186
465	46.45	195.28	51.89	92.93	120.0186
466	69.68	195.28	49.24	79.20	120.0186
467	58.06	610.25	54.04	99.25	120.0186
468	20.90	610.25	66.19	124.64	120.0186
469	46.45	976.40	56.02	80.81	120.0186
470	69.68	366.15	60.78	98.63	120.0186
471	37.16	195.28	54.06	99.40	120.1262
472	27.87	195.28	55.87	104.25	120.2339
473	27.87	195.28	66.45	116.34	120.2339
474	69.68	195.28	62.21	106.26	120.2339
475	83.61	366.15	59.59	96.08	120.3415
476	58.06	610.25	60.17	87.01	120.3415
477	46.45	195.28	51.05	92.93	120.6644
478	97.55	195.28	51.60	83.30	120.6644
479	58.06	366.15	57.45	99.13	120.6644
480	46.45	976.40	60.17	87.01	120.6644
481	58.06	195.28	58.43	103.95	120.7721
482	83.61	195.28	60.17	87.01	120.7721
483	37.16	610.25	47.20	74.35	120.8797
484	27.87	366.15	51.89	92.93	120.8797
485	58.06	195.28	51.89	92.93	120.8797
486	20.90	976.40	66.19	124.64	120.8797
487	148.64	195.28	56.02	80.81	120.8797
488	148.64	195.28	55.90	87.17	120.8797
489	83.61	195.28	47.20	74.35	121.0950
490	58.06	610.25	55.50	90.18	121.0950
491	37.16	610.25	58.43	103.95	121.0950
492	97.55	610.25	56.02	80.81	121.0950
493	20.90	976.40	62.21	106.26	121.0950
494	37.16	366.15	62.21	106.26	121.0950
495	37.16	610.25	49.24	79.20	121.2026
496	46.45	610.25	59.59	96.08	121.2026
497	97.55	195.28	59.59	96.08	121.2026
498	58.06	366.15	62.21	106.26	121.2026
499	69.68	610.25	60.17	87.01	121.2026
500	113.81	195.28	50.95	79.17	121.3103
501	37.16	976.40	59.59	96.08	121.3103
502	37.16	366.15	49.00	87.28	121.4179
503	58.06	366.15	55.77	103.87	121.4179
504	20.90	610.25	66.45	116.34	121.4179
505	58.06	366.15	47.92	84.85	121.6332
506	58.06	366.15	49.97	90.51	121.6332
507	69.68	976.40	50.95	79.17	121.6332
508	20.90	195.28	69.48	125.29	121.6332
509	113.81	195.28	59.59	96.08	121.6332
510	83.61	610.25	50.95	79.17	121.7408
511	46.45	610.25	56.69	92.93	121.7408
512	46.45	366.15	68.89	80.35	121.7408
513	58.06	195.28	63.40	96.22	121.7408
514	46.45	610.25	45.15	77.58	121.8485
515	58.06	366.15	47.08	83.24	121.8485
516	69.68	366.15	55.50	90.18	121.8485
517	58.06	195.28	68.89	80.35	121.8485
518	37.16	610.25	63.40	96.22	121.8485
519	46.45	366.15	47.92	84.85	121.9561
520	46.45	366.15	49.97	90.51	121.9561
521	27.87	976.40	60.78	98.63	121.9561
522	37.16	610.25	68.89	80.35	122.0638
523	46.45	366.15	63.40	96.22	122.0638
524	83.61	195.28	55.50	90.18	122.1714
525	69.68	195.28	57.45	99.13	122.2790
526	130.06	610.25	55.90	87.17	122.2790
527	97.55	366.15	59.59	96.08	122.2790
528	97.55	195.28	60.17	87.01	122.3867
529	97.55	366.15	60.17	87.01	122.3867
530	113.81	195.28	51.60	83.30	122.4943
531	130.06	610.25	56.02	80.81	122.4943
532	46.45	366.15	62.21	106.26	122.4943
533	69.68	976.40	51.60	83.30	122.6020
534	69.68	366.15	47.92	84.85	122.7096
535	69.68	195.28	51.89	92.93	122.7096
536	69.68	366.15	49.97	90.51	122.7096
537	46.45	610.25	55.77	103.87	122.7096
538	130.06	366.15	56.02	80.81	122.7096
539	130.06	366.15	55.90	87.17	122.7096
540	97.55	195.28	45.15	77.58	122.8172
541	69.68	195.28	68.89	80.35	122.8172
542	27.87	976.40	47.20	74.35	122.9249
543	20.90	195.28	53.46	97.78	122.9249
544	27.87	366.15	63.26	116.18	122.9249
545	58.06	610.25	56.69	92.93	123.0325
546	37.16	976.40	58.43	103.95	123.0325
547	58.06	976.40	60.17	87.01	123.0325
548	27.87	195.28	53.46	97.78	123.1402
549	46.45	366.15	47.08	83.24	123.1402
550	20.90	366.15	69.48	125.29	123.1402
551	58.06	976.40	55.90	87.17	123.1402
552	37.16	610.25	62.21	106.26	123.1402
553	83.61	366.15	60.17	87.01	123.1402
554	83.61	366.15	60.78	98.63	123.2478
555	20.90	610.25	55.87	104.25	123.3554
556	83.61	610.25	51.60	83.30	123.3554
557	37.16	366.15	63.26	116.18	123.4631
558	27.87	610.25	62.21	106.26	123.4631
559	69.68	195.28	63.40	96.22	123.4631
560	58.06	195.28	51.05	92.93	123.5707
561	27.87	366.15	54.06	99.40	123.5707
562	46.45	976.40	55.50	90.18	123.5707
563	97.55	195.28	60.78	98.63	123.5707
564	20.90	195.28	68.25	150.03	123.6784
565	37.16	195.28	66.45	116.34	123.6784
566	83.61	195.28	62.21	106.26	123.6784
567	58.06	366.15	49.00	87.28	123.7860
568	20.90	366.15	68.25	150.03	123.8936
569	27.87	366.15	66.45	116.34	123.8936
570	46.45	610.25	68.89	80.35	123.8936
571	20.90	366.15	53.46	97.78	124.0013
572	46.45	366.15	58.43	103.95	124.0013
573	37.16	366.15	51.05	92.93	124.1089
574	58.06	976.40	56.02	80.81	124.1089
575	37.16	976.40	60.78	98.63	124.1089
576	46.45	610.25	60.78	98.63	124.1089
577	148.64	366.15	56.02	80.81	124.2166
578	148.64	366.15	55.90	87.17	124.2166
579	46.45	610.25	63.40	96.22	124.2166
580	69.68	976.40	55.90	87.17	124.3242
581	46.45	366.15	49.00	87.28	124.5395
582	27.87	195.28	66.19	124.64	124.5395
583	37.16	195.28	66.19	124.64	124.5395
584	58.06	976.40	55.50	90.18	124.5395
585	113.81	195.28	60.78	98.63	124.5395
586	58.06	366.15	68.89	80.35	124.5395
587	37.16	195.28	53.46	97.78	124.6471
588	83.61	195.28	56.69	92.93	124.6471
589	69.68	366.15	62.21	106.26	124.6471
590	83.61	610.25	60.17	87.01	124.6471
591	37.16	610.25	47.92	84.85	124.7548
592	37.16	610.25	49.97	90.51	124.7548
593	37.16	195.28	89.43	177.61	124.7548
594	46.45	610.25	47.20	74.35	124.8624
595	20.90	976.40	51.89	92.93	124.8624
596	69.68	366.15	56.69	92.93	124.8624
597	58.06	366.15	49.24	79.20	124.9700
598	58.06	366.15	63.40	96.22	124.9700
599	167.23	195.28	55.90	87.17	125.0777
600	46.45	976.40	55.77	103.87	125.1853
601	20.90	195.28	70.10	143.88	125.1853
602	69.68	610.25	59.59	96.08	125.1853
603	97.55	366.15	60.78	98.63	125.1853
604	83.61	976.40	55.90	87.17	125.2930
605	46.45	976.40	56.69	92.93	125.4006
606	58.06	366.15	58.43	103.95	125.4006
607	69.68	976.40	56.02	80.81	125.4006
608	69.68	366.15	49.00	87.28	125.5082
609	20.90	366.15	70.10	143.88	125.5082
610	97.55	366.15	50.95	79.17	125.5082
611	20.90	976.40	66.45	116.34	125.5082
612	20.90	976.40	54.06	99.40	125.6159
613	83.61	976.40	56.02	80.81	125.6159
614	167.23	195.28	56.02	80.81	125.6159
615	46.45	610.25	57.45	99.13	125.7235
616	20.90	610.25	53.46	97.78	125.8312
617	27.87	976.40	47.08	83.24	125.8312
618	58.06	610.25	55.77	103.87	125.8312
619	58.06	610.25	59.59	96.08	125.8312
620	130.06	195.28	59.59	96.08	125.8312
621	46.45	195.28	53.46	97.78	125.9388
622	46.45	366.15	49.24	79.20	125.9388
623	167.23	366.15	55.90	87.17	125.9388
624	69.68	366.15	68.89	80.35	125.9388
625	37.16	195.28	55.87	104.25	126.0464
626	97.55	195.28	47.20	74.35	126.1541
627	46.45	610.25	47.08	83.24	126.1541
628	69.68	610.25	55.50	90.18	126.1541
629	20.90	610.25	68.25	150.03	126.2617
630	27.87	195.28	69.48	125.29	126.2617
631	83.61	195.28	47.92	84.85	126.3694
632	83.61	195.28	49.97	90.51	126.3694
633	27.87	610.25	51.89	92.93	126.4770
634	97.55	366.15	51.60	83.30	126.4770
635	58.06	610.25	57.45	99.13	126.4770
636	58.06	195.28	53.46	97.78	126.5846
637	27.87	976.40	62.21	106.26	126.5846
638	69.68	366.15	63.40	96.22	126.5846
639	46.45	195.28	54.06	99.40	126.6923
640	167.23	366.15	56.02	80.81	126.6923
641	83.61	366.15	45.15	77.58	126.9076
642	27.87	976.40	47.92	84.85	126.9076
643	83.61	195.28	47.08	83.24	126.9076
644	27.87	976.40	49.97	90.51	126.9076
645	58.06	976.40	56.69	92.93	127.0152
646	69.68	195.28	58.43	103.95	127.0152
647	20.90	610.25	69.48	125.29	127.0152
648	37.16	976.40	68.89	80.35	127.0152
649	46.45	976.40	59.59	96.08	127.2305
650	27.87	610.25	51.05	92.93	127.3381
651	113.81	366.15	59.59	96.08	127.3381
652	37.16	366.15	51.89	92.93	127.4458
653	83.61	610.25	59.59	96.08	127.4458
654	37.16	976.40	63.40	96.22	127.4458
655	58.06	610.25	68.89	80.35	127.5534
656	27.87	366.15	53.46	97.78	127.6610
657	37.16	976.40	47.08	83.24	127.6610
658	69.68	366.15	47.08	83.24	127.7687
659	20.90	976.40	68.25	150.03	127.7687
660	113.81	610.25	59.59	96.08	127.7687
661	69.68	366.15	57.45	99.13	127.8763
662	113.81	195.28	60.17	87.01	127.8763
663	37.16	976.40	45.15	77.58	127.9840
664	37.16	610.25	49.00	87.28	127.9840
665	20.90	195.28	51.89	92.93	127.9840
666	83.61	366.15	62.21	106.26	127.9840
667	97.55	610.25	60.17	87.01	127.9840
668	83.61	366.15	55.50	90.18	128.0916
669	83.61	195.28	68.89	80.35	128.0916
670	37.16	366.15	66.45	116.34	128.1992
671	58.06	610.25	63.40	96.22	128.1992
672	69.68	195.28	51.05	92.93	128.3069
673	113.81	976.40	56.02	80.81	128.3069
674	97.55	195.28	62.21	106.26	128.3069
675	20.90	610.25	70.10	143.88	128.4145
676	27.87	610.25	66.45	116.34	128.5222
677	97.55	610.25	59.59	96.08	128.5222
678	37.16	610.25	51.05	92.93	128.6298
679	83.61	976.40	50.95	79.17	128.6298
680	113.81	366.15	50.95	79.17	128.6298
681	69.68	610.25	56.69	92.93	128.6298
682	46.45	610.25	62.21	106.26	128.6298
683	46.45	976.40	68.89	80.35	128.6298
684	69.68	610.25	68.89	80.35	128.6298
685	27.87	976.40	49.24	79.20	128.7374
686	27.87	366.15	66.19	124.64	128.7374
687	20.90	195.28	73.24	139.26	128.7374
688	20.90	195.28	71.40	142.34	128.7374
689	46.45	610.25	49.24	79.20	128.8451
690	113.81	976.40	55.90	87.17	128.8451
691	130.06	195.28	60.78	98.63	128.8451
692	20.90	366.15	71.40	142.34	128.9527
693	20.90	195.28	71.87	150.07	128.9527
694	46.45	976.40	63.40	96.22	128.9527
695	97.55	366.15	45.15	77.58	129.0604
696	69.68	195.28	53.46	97.78	129.0604
697	20.90	366.15	73.24	139.26	129.0604
698	97.55	976.40	55.90	87.17	129.0604
699	37.16	976.40	62.21	106.26	129.0604
700	83.61	195.28	49.00	87.28	129.1680
701	83.61	195.28	49.24	79.20	129.1680
702	58.06	195.28	54.06	99.40	129.1680
703	27.87	610.25	63.26	116.18	129.1680
704	46.45	195.28	63.26	116.18	129.1680
705	97.55	610.25	50.95	79.17	129.1680
706	83.61	195.28	57.45	99.13	129.1680
707	69.68	610.25	60.78	98.63	129.1680
708	69.68	610.25	63.40	96.22	129.1680
709	83.61	195.28	63.40	96.22	129.1680
710	27.87	366.15	55.87	104.25	129.2756
711	113.81	195.28	62.21	106.26	129.2756
712	46.45	976.40	57.45	99.13	129.3833
713	46.45	610.25	47.92	84.85	129.4909
714	46.45	610.25	49.97	90.51	129.4909
715	97.55	976.40	56.02	80.81	129.4909
716	148.64	195.28	59.59	96.08	129.4909
717	27.87	976.40	49.00	87.28	129.7062
718	69.68	366.15	49.24	79.20	129.7062
719	27.87	976.40	63.26	116.18	129.7062
720	37.16	610.25	63.26	116.18	129.7062
721	37.16	366.15	66.19	124.64	129.7062
722	83.61	976.40	51.60	83.30	129.7062
723	113.81	366.15	51.60	83.30	129.7062
724	97.55	195.28	55.50	90.18	129.7062
725	148.64	610.25	56.02	80.81	129.7062
726	148.64	610.25	55.90	87.17	129.7062
727	97.55	195.28	68.89	80.35	129.7062
728	97.55	366.15	68.89	80.35	129.7062
729	97.55	366.15	62.21	106.26	129.8138
730	58.06	366.15	51.89	92.93	129.9215
731	37.16	195.28	69.48	125.29	129.9215
732	58.06	610.25	60.78	98.63	129.9215
733	58.06	195.28	76.00	110.08	129.9215
734	20.90	195.28	63.33	116.37	130.0291
735	20.90	976.40	70.10	143.88	130.0291
736	83.61	366.15	47.20	74.35	130.1368
737	37.16	366.15	54.06	99.40	130.1368
738	27.87	366.15	69.48	125.29	130.1368
739	37.16	976.40	63.26	116.18	130.2444
740	113.81	366.15	60.78	98.63	130.2444
741	20.90	195.28	76.00	110.08	130.2444
742	69.68	976.40	60.17	87.01	130.2444
743	113.81	366.15	60.17	87.01	130.2444
744	148.64	366.15	125.40	156.10	130.2444
745	83.61	610.25	60.78	98.63	130.3520
746	37.16	195.28	76.00	110.08	130.3520
747	46.45	610.25	58.43	103.95	130.4597
748	46.45	366.15	51.89	92.93	130.5673
749	46.45	976.40	60.78	98.63	130.5673
750	37.16	976.40	49.24	79.20	130.6750
751	20.90	195.28	73.49	143.88	130.6750
752	97.55	610.25	51.60	83.30	130.7826
753	83.61	366.15	56.69	92.93	130.7826
754	58.06	976.40	57.45	99.13	130.7826
755	58.06	976.40	68.89	80.35	130.7826
756	83.61	366.15	68.89	80.35	130.7826
757	97.55	195.28	63.40	96.22	130.7826
758	97.55	366.15	63.40	96.22	130.7826
759	130.06	610.25	125.40	156.10	130.7826
760	69.68	366.15	51.89	92.93	130.8902
761	113.81	610.25	60.78	98.63	130.8902
762	97.55	366.15	47.20	74.35	130.9979
763	27.87	976.40	66.45	116.34	130.9979
764	130.06	976.40	55.90	87.17	130.9979
765	130.06	366.15	59.59	96.08	130.9979
766	20.90	366.15	76.00	110.08	130.9979
767	113.81	195.28	45.15	77.58	131.1055
768	20.90	610.25	73.24	139.26	131.1055
769	20.90	366.15	71.87	150.07	131.1055
770	130.06	610.25	59.59	96.08	131.1055
771	46.45	976.40	45.15	77.58	131.2132
772	20.90	976.40	69.48	125.29	131.2132
773	58.06	610.25	45.15	77.58	131.3208
774	27.87	195.28	68.25	150.03	131.3208
775	37.16	195.28	68.25	150.03	131.3208
776	167.23	610.25	55.90	87.17	131.3208
777	130.06	195.28	60.17	87.01	131.3208
778	58.06	976.40	63.40	96.22	131.3208
779	83.61	366.15	63.40	96.22	131.3208
780	20.90	610.25	71.40	142.34	131.4284
781	130.06	976.40	56.02	80.81	131.4284
782	97.55	610.25	60.78	98.63	131.4284
783	27.87	195.28	76.00	110.08	131.4284
784	97.55	195.28	47.92	84.85	131.5361
785	97.55	195.28	49.97	90.51	131.5361
786	20.90	976.40	55.87	104.25	131.5361
787	46.45	195.28	76.00	110.08	131.5361
788	20.90	976.40	53.46	97.78	131.6437
789	37.16	976.40	47.20	74.35	131.7514
790	69.68	610.25	45.15	77.58	131.7514
791	58.06	610.25	58.43	103.95	131.7514
792	46.45	366.15	63.26	116.18	131.8590
793	58.06	195.28	63.26	116.18	131.8590
794	113.81	610.25	50.95	79.17	131.8590
795	69.68	976.40	55.50	90.18	131.8590
796	113.81	195.28	55.50	90.18	131.8590
797	97.55	195.28	56.69	92.93	131.8590
798	46.45	195.28	66.45	116.34	131.8590
799	167.23	610.25	56.02	80.81	131.8590
800	58.06	976.40	59.59	96.08	131.9666

#### Detection of outliers

Outlier identification using the Interquartile Range (IQR) method is a well-established approach for detecting anomalies in a dataset^44^. This method revolves around analyzing the spread of the data by calculating key percentiles. Specifically, the IQR is the range between the 25th percentile, also called as the first quartile (Q1), and the 75th percentile, called as the third quartile (Q3). The IQR effectively captures the middle 50% of the data, which accounts to the central tendency of the dataset. Equation 1 is used to calculate the IQR.

1 $$\:\text{IQR}=\text{Q}3-\text{Q}1$$

Once the IQR is calculated, outlier detection thresholds are defined based on extending 1.5 times the IQR beyond Q1 and Q3. These thresholds are represented by the lower and upper boundaries. To determine the lower bound, subtract 1.5 times the IQR from Q1, while the upper bound is calculated by adding 1.5 times the IQR to Q3. The respective equations (Eqs. 2–3) are:

2 $$\:\text{Lower\:Bound}=\text{Q}1-1.5\times\:\text{IQR}$$

3 $$\:\text{Upper\:Bound}=\text{Q}3+1.5\times\:\text{IQR}$$

Any data points that fall below the lower bound or above the upper bound are classified as outliers. These outliers are considered to be extreme values that deviate significantly from the rest of the data. Identifying and removing outliers using this method helps in preventing skewing of the analysis or modeling results, as outliers can disproportionately affect calculations like means or regression coefficients.

**Fig. 2:**
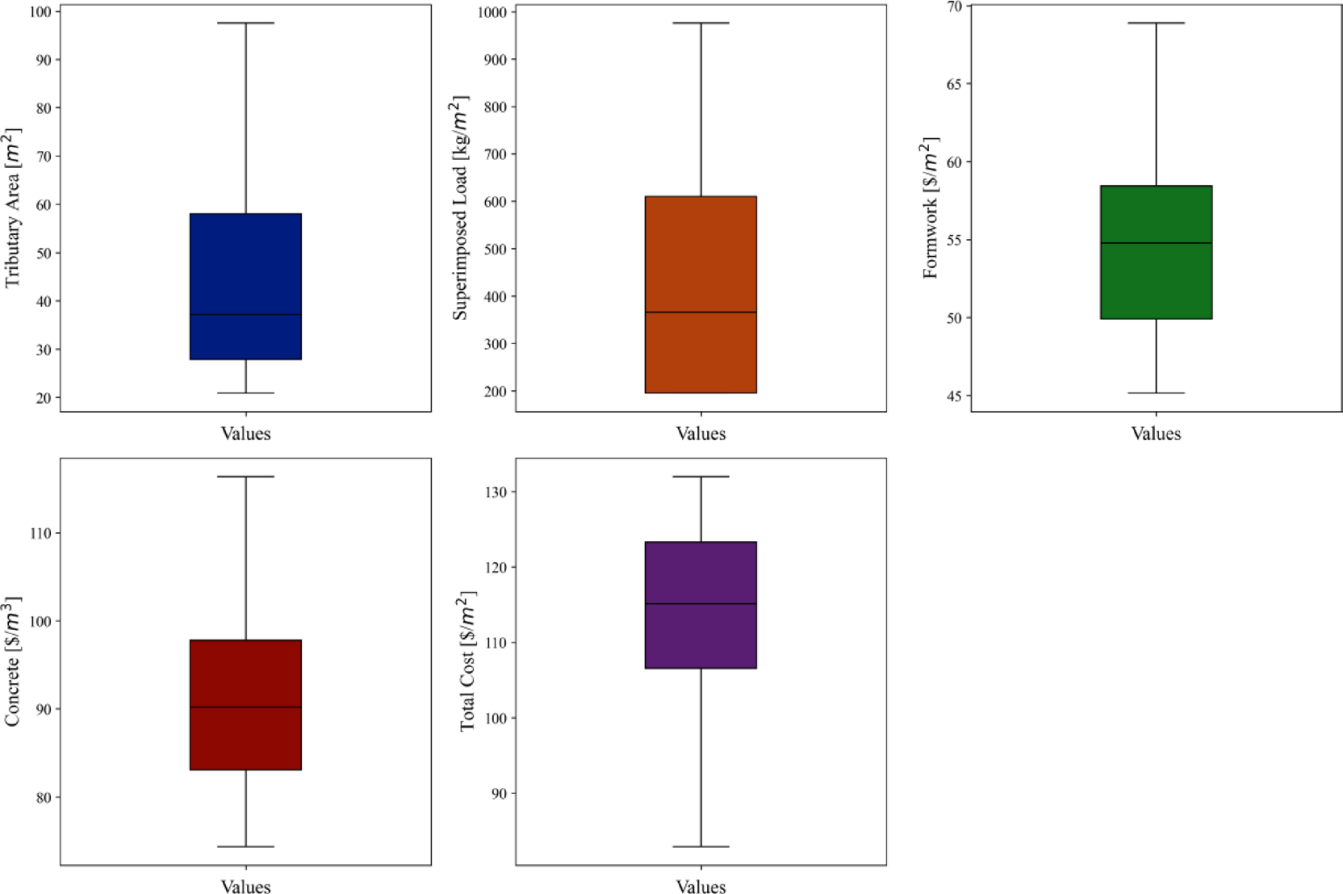
Boxplots for detecting outliers in the collected datasets.

In this study, out of the 800 data points collected for the prediction of the total construction cost of concrete slabs, the outlier detection process using the IQR method identified and removed several extreme data points to create a “cleaned” dataset. After removing these outliers, 704 clean data points remained. This cleaning process ensures that the resulting dataset is free from extreme values, providing a more accurate representation of the true characteristics of the data. To visually confirm that the outliers have been effectively removed, a boxplot illustrating the distribution of the cleaned data is shown in Fig. 2. This figure demonstrates that there are no remaining outliers, ensuring the integrity of the dataset for subsequent analysis, modeling, and insights.

#### Statistical summary

Table 2 shows the descriptive statistics for various parameters measured in a dataset, highlighting the range, central tendency, dispersion, and distribution characteristics of the variables. The table presents descriptive statistics for a dataset that includes several parameters related to construction costs and load factors. For each of the parameters, key statistical measures such as minimum, maximum, mean, median, standard deviation (Std. Dev.), kurtosis, and skewness are provided. These metrics offer insights into the central tendency, variability, and distribution shape of the data.

**Table 2:** Descriptive statistics of collected dataset.

Parameters	Min	Max	Mean	Median	Std. Dev.	Kurtosis	Skewness
Tributary Area ($/m^2^)	20.90	97.55	45.91	37.16	21.89	-0.275	0.789
Superimposed Load (kg/m^2^)	195.28	976.40	475.23	366.15	276.68	-0.744	0.732
Formwork ($/m^2^)	45.15	68.89	54.50	54.78	5.74	-0.464	0.442
Concrete ($/m^3^)	74.35	116.37	90.50	90.18	9.94	-0.162	0.562
Total Cost ($/m^2^)	82.88	131.97	114.31	115.12	11.22	-0.495	-0.458

The first parameter (Tributary Area, $/m^2^), shows a minimum value of 20.90 and a maximum value of 97.55, indicating a wide range of data. The mean and median values are 45.91 and 37.16, respectively, suggesting that the data may be slightly skewed towards higher values, as the mean is larger than the median. The standard deviation is 21.89, reflecting considerable variation within the data. The kurtosis is slightly negative at − 0.275, which suggests that the distribution is less peaked than a normal distribution, while the positive skewness of 0.789 indicates a moderate skew towards higher values. For the second parameter (Superimposed Load, kg/m^2^), the minimum value is 195.28, and the maximum is 976.40, with a mean of 475.23 and a median of 366.15. This difference between the mean and median highlights a potential right-skewed distribution, confirmed by the positive skewness value of 0.732. The standard deviation is 276.68, pointing to a high degree of variability in the dataset. The kurtosis is − 0.744, indicating that the distribution has lighter tails than a normal distribution, which suggests fewer extreme values than expected.

The 3rd parameter (Formwork, $/m^2^) has a narrower range, with a minimum of 45.15 and a maximum of 68.89. The mean and median are close to each other, at 54.50 and 54.78, respectively, implying that the data is fairly symmetric. This is supported by the relatively low skewness value of 0.442. The standard deviation is 5.74, which indicates moderate variability in the formwork costs. The kurtosis value is − 0.464, showing that the distribution is slightly flatter than normal, but there are no significant outliers or extreme values. For the 4th parameter (Concrete, $/m^3^), the minimum cost is 74.35, and the maximum is 116.37, with a mean of 90.50 and a median of 90.18. The closeness between the mean and median indicates that the data is approximately symmetric, and the skewness of 0.562 supports this observation. The standard deviation of 9.94 suggests moderate variability in concrete costs. The kurtosis is − 0.162, implying a distribution that is slightly flatter than normal but not significantly different.

Finally, Total Cost ($/m^2^) exhibits a minimum value of 82.88 and a maximum value of 131.97, with a mean of 114.31 and a median of 115.12. The standard deviation is 11.22, showing moderate variation in the total cost. The kurtosis is − 0.495, which points to a somewhat flatter distribution compared to the normal distribution. The skewness is − 0.458, indicating a slight skew towards lower total costs, although this skew is not pronounced. Overall, the dataset presents moderate variability across the different parameters, with most distributions displaying slight skewness and relatively low kurtosis, suggesting they are generally close to normal but with minor deviations. This analysis highlights the range and distribution characteristics of the various construction-related factors in the dataset.

#### Histograms

**Fig. 3:**
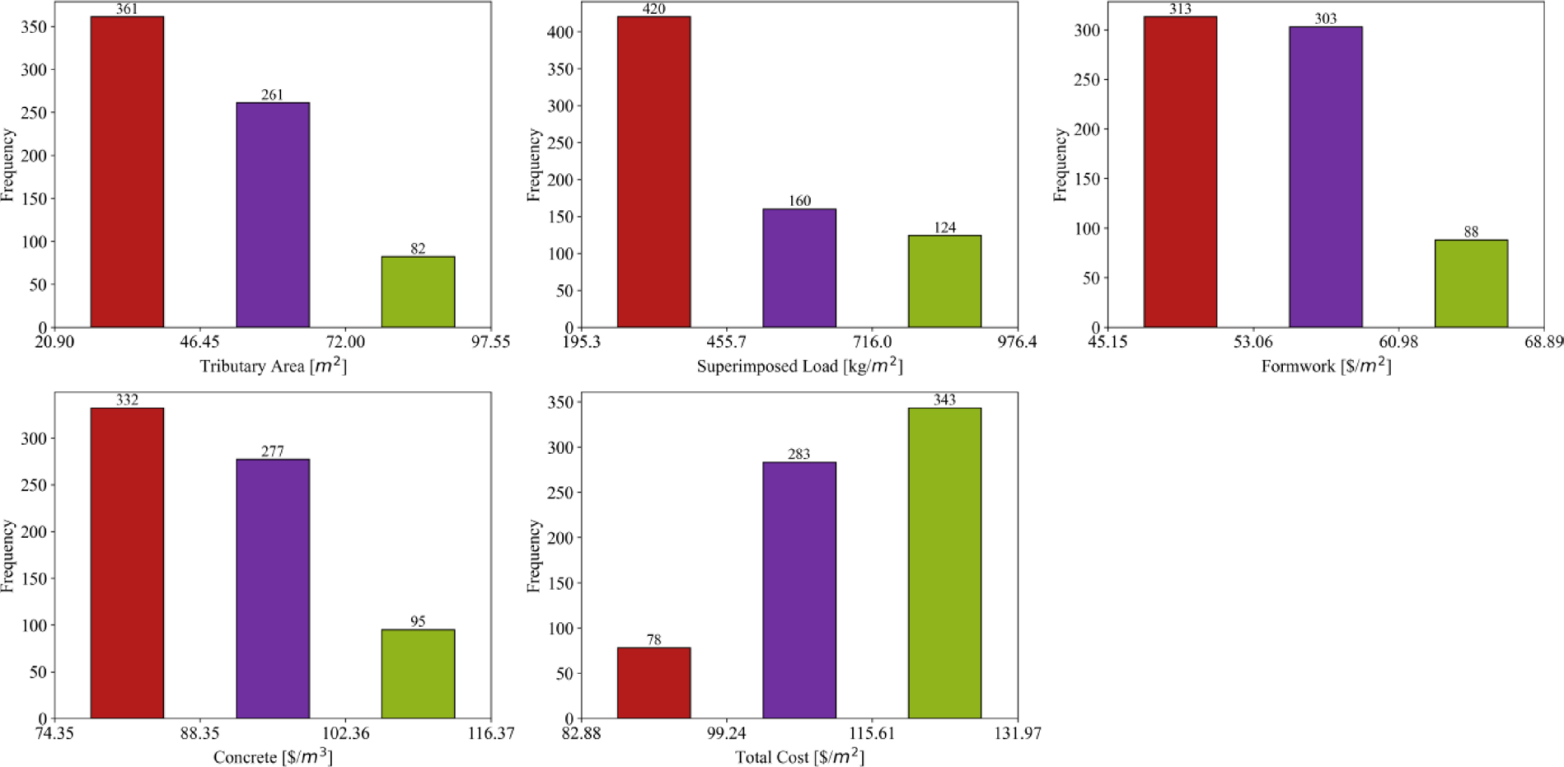
Histograms of the collected datasets.

Figure 3 presents a series of histograms that illustrate the frequency distributions of various parameters measured in the dataset. These histograms provide a visual representation of the data, allowing us to observe the distribution patterns and the concentration of values within specific ranges for each parameter. In the first bar chart for Tributary Area ($/m^2^), the majority of the data points are concentrated in the lower value ranges, with 361 occurrences in the range of approximately 20.90 to 46.45 $/m^2^. This is followed by 261 occurrences between 46.45 and 72.00 $/m^2^, and 82 occurrences in the highest range, between 72.00 and 97.55 $/m^2^. This pattern suggests that most data points for the tributary area fall in the lower value ranges, with fewer instances of higher values, corresponding to the positive skewness noted in the descriptive statistics.

The second bar chart for Superimposed Load (kg/m^2^) follows a similar trend, where the most frequent values are found in the lower range between 195.3 and 455.7 kg/m^2^, with 420 occurrences. The middle range (455.7–716.0 kg/m^2^) has 160 occurrences, and the highest range (716.0–976.4 kg/m^2^) shows 124 occurrences. This distribution is reflective of the positive skew in the dataset, as higher values of superimposed load are less frequent. In the third chart for Formwork ($/m^2^), the data appears more evenly distributed across the lower and middle value ranges. The range from 45.15 to 53.06 $/m^2^ has 313 occurrences, while the next range (53.06 to 60.98 $/m^2^) has 303 occurrences. The highest range (60.98–68.89 $/m^2^) has fewer data points, with 88 occurrences. This indicates a relatively symmetric distribution of values, consistent with the minimal skewness observed in the statistical summary.

The fourth chart for Concrete ($/m^3^) shows a clear concentration of data points in the lower value range, with 332 occurrences between 74.35 and 88.35 $/m^3^. The middle range (88.35–102.36 $/m^3^) has 277 occurrences, while the highest range 102.36–116.37 $/m^3^) has 95 occurrences. This suggests that most of the concrete costs fall in the lower range, and the distribution has a slight positive skew. Finally, the fifth chart for Total Cost ($/m^2^) shows a higher concentration of data points in the middle range, with 343 occurrences between 115.61 and 131.97 $/m^2^. The lower range (82.88–99.24 $/m^2^) has 78 occurrences, while the middle range (99.24 to 115.61 $/m^2^) shows 283 occurrences. The distribution indicates that total costs are fairly concentrated around the median values, with fewer data points at the extremes. Overall, the figure provides a clear visual representation of how the values for each parameter are distributed, with some parameters showing more skewed distributions (Tributary Area, Superimposed Load) and others displaying more symmetry (Formwork, Total Cost).

#### Hexbin plots

**Fig. 4:**
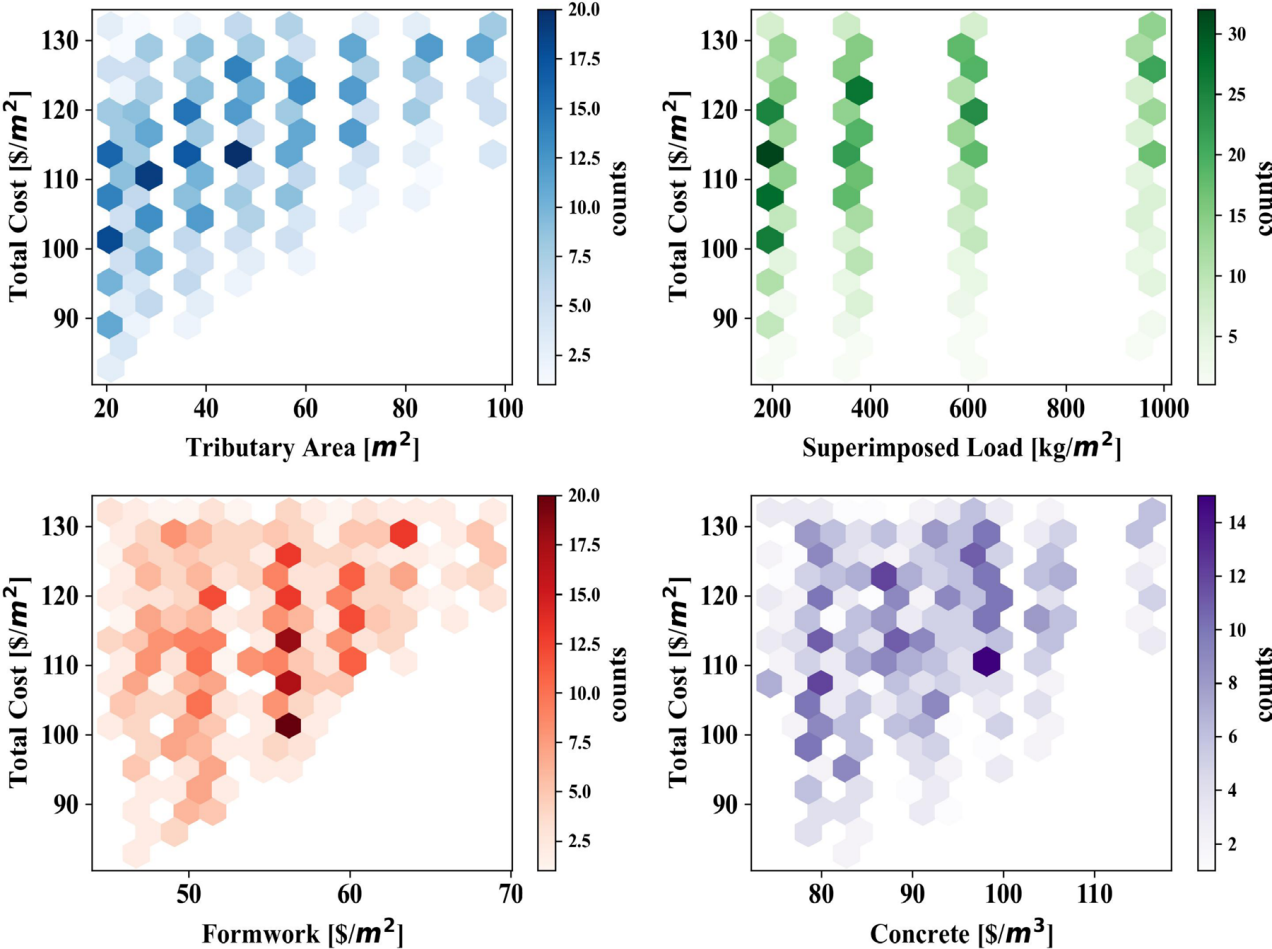
Hexbins plots of the collected datasets.

Figure 4 illustrates hexbin plots representing the relationships between total cost per square meter and the four input variables: tributary area, superimposed load, formwork cost, and concrete cost. Each hexbin plot provides a visualization of data density within specific ranges of these variables, with color intensity corresponding to the frequency of data points. In the top-left plot, the relationship between total cost per square meter and tributary area is depicted. The data shows a concentration of costs ranging from $90/m² to $130/m², with tributary areas clustering predominantly between 20 and 100 m^2^. The darker hexagons indicate regions where these cost-area combinations are more frequent, highlighting the distribution of construction projects or designs with typical tributary areas and associated costs. The top-right plot examines the impact of superimposed load on total cost per square meter. Here, costs are distributed across a superimposed load range from 200 to 1000 kg/m^2^, with clear bands of high-frequency data. The consistent vertical alignment of the darker hexagons suggests discrete categories or design constraints influencing superimposed loads in this dataset. The bottom-left plot focuses on the relationship between formwork cost and total cost per square meter. It shows a broad distribution of formwork costs between $50/m^2^ and $70/m^2^, with concentrations in regions where total costs range from $100/m^2^ to $130/m^2^. The denser areas of the plot indicate that variations in formwork costs contribute significantly to the overall construction cost.

Finally, the bottom-right plot captures the relationship between concrete cost and total cost per square meter. This plot reveals a more dispersed pattern, with concrete costs spanning $80/m^2^ to $110/m^2^. The areas of higher density suggest that specific concrete cost levels correlate more frequently with total costs in the $100/m² to $120/m² range, reflecting common material and pricing choices. The plots demonstrate clear patterns and dense clusters for each variable in relation to the total cost per square meter. The structured relationships and well-distributed data points across different ranges of tributary area, superimposed load, formwork cost, and concrete cost suggest that the dataset is comprehensive and exhibits meaningful variability. These characteristics make it suitable for further ML analysis. The density and distribution of the data provide a solid foundation for training predictive models, enabling accurate identification of cost-driving factors and generating reliable predictions for construction cost optimization.

#### Scatter pair plots

**Fig. 5:**
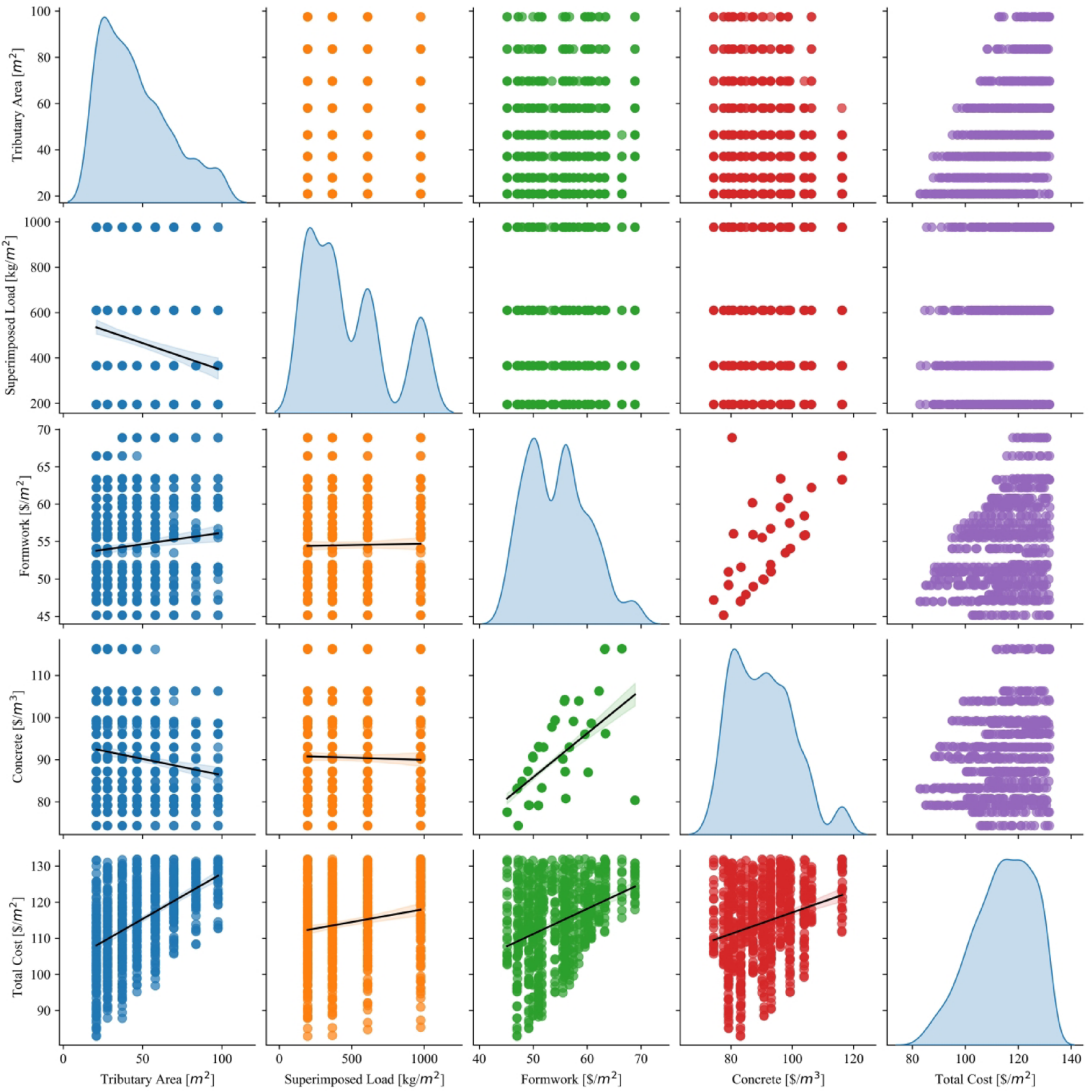
Scatter pair plots of the collected datasets.

Figure 5 presents a matrix of scatter pair plots with trend lines for the variables in the dataset. Each diagonal of the matrix contains the distribution of each parameter, while the off-diagonal plots represent pairwise scatterplots with regression lines showing the trends between two variables. This visualization allows for a comprehensive view of both the individual distribution of variables and the correlation patterns between them. For the Tributary Area parameter, its distribution shows a heavily right-skewed shape, which indicates that most of the values are concentrated towards the lower end, while only a few observations are found in higher ranges. When paired with other parameters, the scatterplots show some degree of variation. There seems to be a weak positive linear relationship with Total Cost, as indicated by the regression line in the scatterplot between these two variables. However, the other pairwise plots do not show a strong relationship between Tributary Area and other parameters like Superimposed Load, Formwork, or Concrete.

The Superimposed Load parameter shows a multimodal distribution, suggesting the presence of clusters or distinct groups within the data. In the scatterplots with other parameters, the relationship with Total Cost is somewhat weak but still positive, as seen by the slightly upward-sloping regression line. This means that higher superimposed loads are generally associated with higher total costs, though the relationship is not strong. Additionally, the scatterplot with Formwork does not show any noticeable correlation, implying that changes in superimposed load do not significantly affect the formwork cost. The Formwork parameter distribution is more uniform and balanced, reflecting less skew compared to other variables. The pairwise plots with Concrete and Total Cost display moderate positive correlations, particularly with Total Cost, where the regression line shows a clear upward trend. This suggests that increases in formwork costs are associated with corresponding increases in total construction costs. The plot between Formwork and Concrete shows a similar but weaker pattern, indicating that formwork costs may also be slightly associated with concrete costs.

The Concrete parameter displays a relatively symmetric distribution. Its scatterplot with Total Cost reveals one of the strongest positive relationships in the dataset, with a clear upward trend in the regression line. This indicates that higher concrete costs are strongly associated with higher total costs, which makes sense in the context of construction projects where concrete forms a significant part of the total expenditure. The scatterplots between concrete and other parameters, such as Tributary Area or Superimposed Load, show weaker or non-existent correlations. Finally, the Total Cost parameter distribution is shown in the far-right diagonal and follows a slightly right-skewed pattern. The pairwise scatterplots confirm that total cost is moderately to strongly correlated with both formwork and concrete costs, as reflected in the positive slopes of the regression lines. The relationship between Total Cost and the other parameters, such as Tributary Area and Superimposed Load, is positive but weaker in comparison to its relationships with formwork and concrete.

#### Correlation analysis

**Fig. 6:**
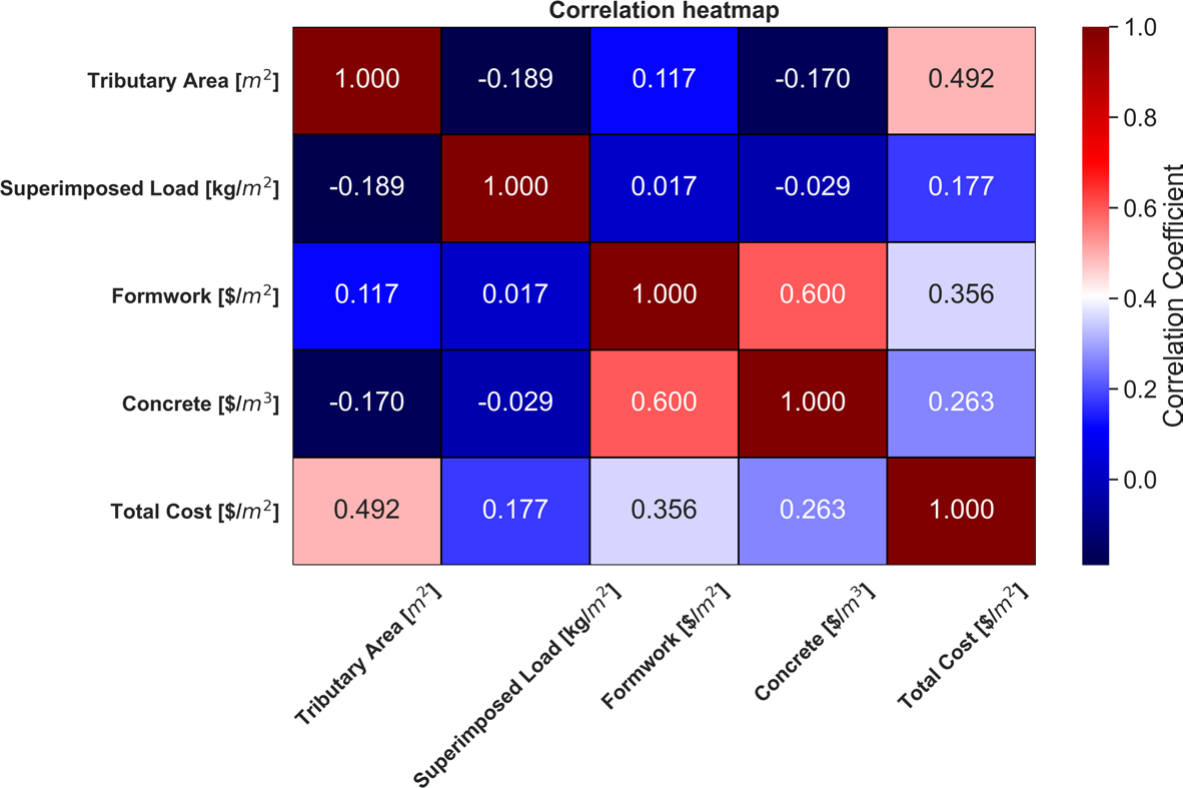
Correlation heatmap illustrating the relationship between the variables.

Figure 6 presents the correlation coefficients for the input and output variables. The Tributary Area shows a moderate positive correlation with Total Cost (0.492), indicating that as the tributary area increases, the total cost tends to rise. It has a weak negative correlation with both Superimposed Load (− 0.189) and Concrete (− 0.170), suggesting a slight inverse relationship with these parameters. The correlation with Formwork is weakly positive (0.117). Superimposed Load displays very weak correlations with other parameters. Its relationship with Total Cost is weakly positive (0.177), while its correlations with Formwork (0.017) and Concrete (− 0.029) are near zero, indicating almost no linear relationship with these variables. Formwork shows a moderate positive correlation with Concrete (0.600), implying a notable association between the two variables. The correlation with Total Cost is also moderately positive (0.356), suggesting that higher formwork costs are linked to higher total costs. Concrete exhibits a moderate positive correlation with Formwork (0.600) and a weaker positive relationship with Total Cost (0.263). This suggests that increases in concrete costs are associated with higher total costs, but the relationship is less strong compared to formwork. Total Cost has the strongest correlation with Tributary Area (0.492) and a moderate correlation with Formwork (0.356), followed by a weaker positive correlation with Concrete (0.263). The correlations with Superimposed Load are weak (0.177), reflecting that it has less impact on the overall cost compared to other factors.

### Data preprocessing

#### Multicollinearity and hypothesis analyses

Min–max normalization can help mitigate issues like multicollinearity in regression analysis^45^. This technique is applied using the formula in Eq. (4), as shown below:

4 $$\:{X}_{n}=\:\frac{X-{X}_{min}}{{X}_{max}-{X}_{min}}$$

In this equation, *X*_*min*_ and *X*_*max*_ represent the minimum and maximum values of the variable, respectively. *X*_*n*_​ denotes the normalized value of the variable, and *X* refers to the original variable value being adjusted. This process scales the data to a range between 0 and 1, with the minimum value set to 0, the maximum value to 1, and all other values proportionally distributed within this range. Multicollinearity occurs when predictor variables are highly correlated, making it hard to assess their individual effects. It’s measured using the Variance Inflation Factor (VIF), where values below 2.5 indicate weak multicollinearity and values above 10 suggest problematic multicollinearity^46,47^. ANOVA and Z-tests were performed using SPSS to evaluate the impact of input variables on the dependent variable. ANOVA partitions the total variance into regression sum of squares and residual sum of squares, with the total sum of squares representing their sum. The model’s significance is tested using the F-statistic, and a p-value less than 0.05 indicates statistical significance. The Z-test assesses if sample means differ from a hypothesized value, with a p-value below 0.05 suggesting a significant difference.

#### Sensitivity analysis

Sensitivity Analysis (SA) is a crucial technique for identifying the most influential input variables in predictive or forecasting models^48^. It can be categorized into linear and nonlinear types. This study employs global cosine amplitude sensitivity analysis to determine the key parameters significantly affecting output predictions. The mathematical expression for cosine amplitude sensitivity analysis is given by Eq. (5).

5 $$\:SA=\frac{{\sum\:}_{c=1}^{n}\left({X}_{ic}\cdot{X}_{jk}\right)}{\sqrt{{\sum\:}_{c=1}^{n}{{X}_{ic}}^{2}}\cdot\sqrt{{\sum\:}_{c=1}^{n}{{X}_{jk}}^{2}}}$$

where *X*_*ic*_ is the input parameters, and is *X*_*jk*_ output parameter. An SA value close to one suggests that the independent variable has a strong impact on the output.

### Overview of catboost model

CatBoost, short for “Categorical Boosting,” is an open-source gradient boosting library developed by Yandex^49^. It efficiently handles categorical features without extensive preprocessing, utilizing symmetric decision trees for faster training and prediction times. With GPU acceleration, CatBoost reduces training times for large datasets and often outperforms other gradient boosting implementations in accuracy and speed. Its ordered boosting technique minimizes overfitting, enhancing generalization to unseen data. However, CatBoost may require significant memory resources, especially for large datasets with high-cardinality categorical features, and training can be computationally intensive with default hyperparameters. Despite these considerations, CatBoost has been applied across various domains, including recommendation systems, fraud detection, medical data analysis, and sales forecasting^50^.

### Description of hybrid catboost models

**Fig. 7:**
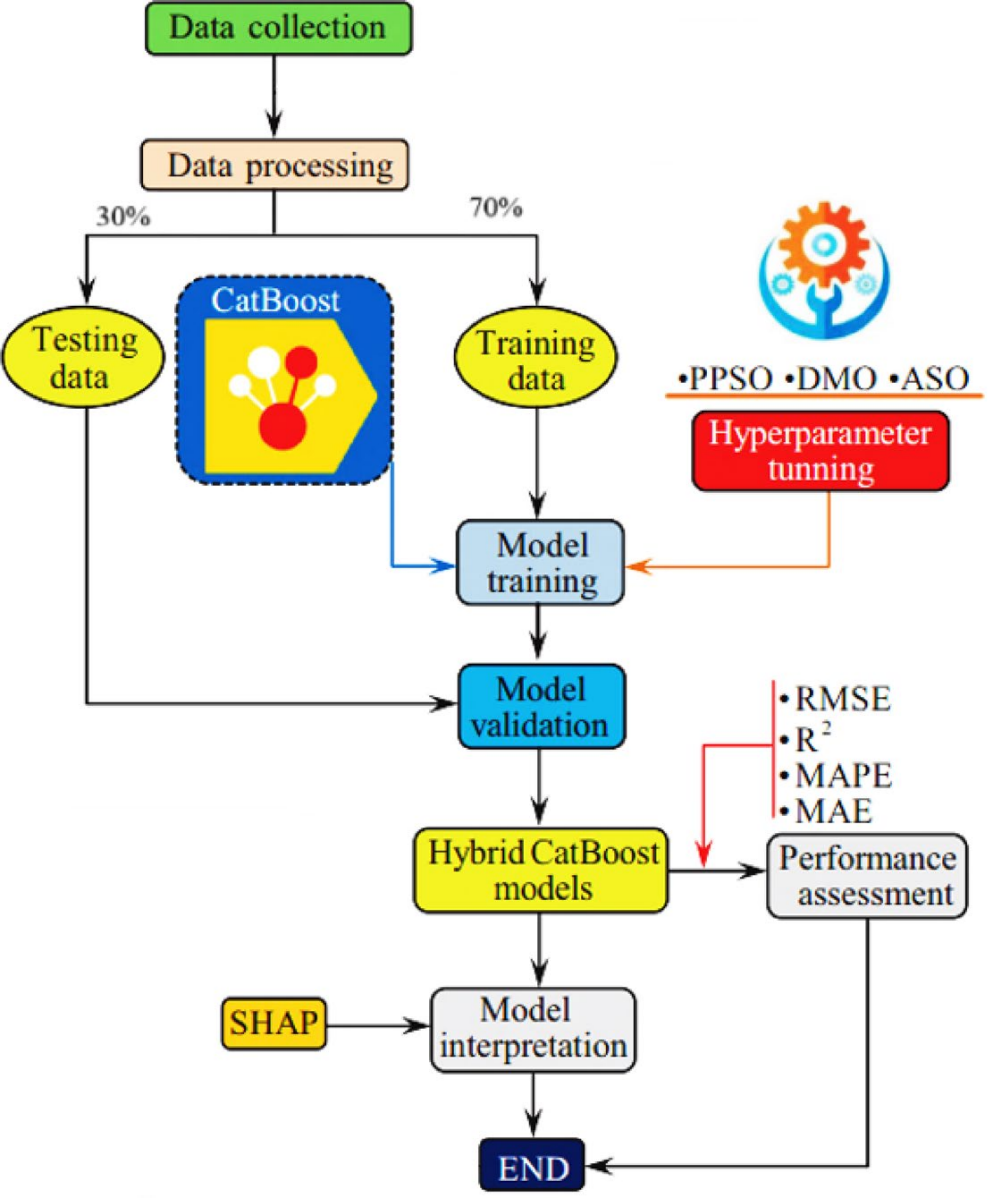
Adopted prediction workflow in this study.

Figure 7 shows the methodological approach adopted in this study. Firstly, the database was collected, then it was randomly divided into 70% for training and 30% for testing stage. After that, using the training data, the hyperparameters of the CatBoost model were optimized using three different optimization algorithms: Phasor Particle Swarm Optimization (PPSO), Dwarf Mongoose Optimization (DMO), and Atom Search Optimization (ASO), and hybrid models (PPSO-CatBoost, DMO-CatBoost, and ASO-CatBoost) were obtained. The optimization process is computationally demanding. On average, PPSO-CatBoost required approximately 3.5–4 h, DMO-CatBoost about 4–4.5 h, and ASO-CatBoost around 4.5–5 h to complete. These times reflect the need to balance model accuracy and runtime efficiency, especially for large-scale or real-time applications.

The selection of PPSO, DMO, and ASO as optimization techniques is grounded in their proven effectiveness in navigating complex and high-dimensional search spaces, as well as their robustness against becoming trapped in local optima and their flexibility across diverse optimization scenarios^51–57^. These hybrid models leverage the strengths of both metaheuristic search and gradient boosting to achieve more refined hyperparameter configurations. Unlike traditional algorithms such as Particle Swarm Optimization (PSO) and Genetic Algorithm (GA), which often require extensive tuning and may suffer from premature convergence or slow exploitation phases, PPSO, DMO, and ASO demonstrate superior convergence behavior, adaptability, and enhanced balance between exploration and exploitation. This makes them particularly suitable for tuning ML models where the search space is non-linear, multi-modal, and sensitive to parameter settings.

#### PPSO-CatBoost

The original Particle Swarm Optimization (PSO) is a metaheuristic optimization method inspired by animal swarm behavior. It optimizes nonlinear functions by tracking particle movement and minimizing assumptions. In PSO, each particle is defined by its position, velocity, and fitness, and follows both a global optimal and an individual optimal trajectory. The algorithm uses stochastic variables to adjust the particle velocities towards personal best ($\:pbest$) and global best ($\:gbest$) positions^58^. In PPSO, the phasor angles *θ* are used instead of constants and arbitrary integers. Additionally, in PPSO, the inertia weight is set to zero. A key advantage of the PPSO algorithm over other methods is its improved efficiency in optimization, even as the problem size increases. In the PPSO algorithm, the position and velocity vectors are denoted by $\:{X}_{ij}$ and $\:{V}_{ij}$ (Eqs. 6, 7), respectively, with the velocity vector updated as shown in Eq. (8). Equations (9, 100) were selected after evaluating and testing different functions for PPSO on real test functions by Ghasemi et al.^59^. Subsequently, the updated position of the particle is calculated by adding the velocity vector to its current position, as expressed in Eq. (11):

6 $$\:{X}_{ij}=\left[{x}_{i1},{x}_{i2},\dots\:,{x}_{ij}\right]$$

7 $$\:{V}_{ij}=\left[{v}_{i1},{v}_{i2},\dots\:,{v}_{ij}\right]$$

8 $$\:{V}_{ij}^{t+1}=p\left({\theta\:}_{ij}^{t}\right)\times\:\left(p{best}_{ij}-{X}_{ij}^{t}\right)+g\left({\theta\:}_{ij}^{t}\right)\cdot\:\times\:\left(g{best}_{ij}-{X}_{ij}^{t}\right)$$

9 $$\:p\left({\theta\:}_{ij}^{t}\right)={\left|\text{cos}{\theta\:}_{ij}^{t}\right|}^{2\times\:\text{sin}{\theta\:}_{ij}^{t}}$$

10 $$\:g\left({\theta\:}_{ij}^{t}\right)={\left|\text{sin}{\theta\:}_{ij}^{t}\right|}^{2\times\:\text{cos}{\theta\:}_{ij}^{t}}$$

11 $$\:{X}_{ij}^{t+1}={X}_{ij}^{t}+{V}_{ij}^{t+1}$$

#### DMO-CatBoost

DMO algorithm is a newly developed swarm-based metaheuristic method inspired by the behavioral patterns of dwarf mongooses. Unlike other species that create nests for their offspring, dwarf mongooses display a distinctive behavior by relocating their young from one sleeping mound to another, consciously avoiding previously explored areas. This behavior is mimicked in the DMO algorithm, which aims to improve optimization by continuously exploring new solutions and avoiding repetition. The DMO model integrates three distinct behavioral layers that correspond to roles observed in mongoose life: alpha, scouts, and babysitters. In the DMO framework, a cohesive group of mongooses concurrently engages in both exploration of new mounds and foraging activities. As the alpha group initiates its foraging behavior, it simultaneously explores new mounds, intending to move to these locations once the babysitter change criterion is met^60^. The optimization procedure within the DMO begins by initializing the candidate population (*X*), representing dwarf mongooses^60^. This population is generated by adhering to the problem’s boundary conditions, defined by the lower and upper bounds, as specified in Eq. (12).

12 $$X = \left[ {\begin{array}{*{20}c} {x_{{1,1}} } & \cdots & {x_{{1,d}} } \\ \vdots & \ddots & \vdots \\ {x_{{n,1}} } & \cdots & {x_{{n,d}} } \\ \end{array} } \right]$$

where *X* is the set of current candidate populations, *n* and *d* denote the population size and the dimension of the problem, respectively. Then, the fitness value is calculated for each solution and the probability of each individual being alpha is calculated using Eq. (13).

13 $$\:\alpha\:=\frac{Fi{t}_{i}}{\sum\:_{i=1}^{n}Fi{t}_{i}}$$

The calculation of the sleeping mound is determined by Eq. (14), and subsequently, the average value of the sleeping mound is computed using Eq. (15).

14 $$\:{sm}_{i}=\frac{Fi{t}_{i+1}-Fi{t}_{i}}{max\left\{Fi{t}_{i+1},Fi{t}_{i}\right\}}$$

15 $$\:\phi\:=\frac{\sum\:_{i=1}^{n}s{m}_{i}}{n}$$

The movement vector is calculated using Eq. (16). After all, the next position of the scout mongoose is determined using Eq. (17), and these operations are repeated for the maximum number of iterations to obtain the best solution.

16 $$\:\overrightarrow{M}={\sum\:}_{i=1}^{n}\frac{{X}_{i}\times\:s{m}_{i}}{{X}_{i}}$$

17 $$\:{X}_{i+1}=\left\{\begin{array}{c}{X}_{i}-{\left(1-\frac{iter}{{Max}_{iter}}\right)}^{\left(\frac{2\times\:iter}{{Max}_{iter}}\right)}\times\:phi\times\:rand\times\:\left[{X}_{i}-\overrightarrow{M}\right]\:if\:{\phi\:}_{i+1}>{\phi\:}_{i}\\\:{X}_{i}+{\left(1-\frac{iter}{{Max}_{iter}}\right)}^{\left(\frac{2\times\:iter}{{Max}_{iter}}\right)}\times\:phi\times\:rand\times\:\left[{X}_{i}-\overrightarrow{M}\right]\:else\end{array}\right.\:$$

where *phi* and *rand* are a uniformly distributed random number [− 1, 1] and a random number between [0, 1], respectively.

#### ASO-CatBoost

Atom Search Optimization (ASO) is a novel physics-based metaheuristic algorithm designed to solve global optimization problems. The ASO algorithm operates by simulating basic molecular dynamics, specifically focusing on the interactions between atoms and their neighboring atoms. These interactions are modeled by considering both attractive and repulsive forces from neighboring atoms as well as constraint forces from the best atom (i.e., the best solution). The algorithm creates a model of atomic motion based on these forces^61^. The primary objective of the ASO algorithm is to optimize the value of the fitness function by determining the atom’s velocity and position, while accounting for its mass and the forces acting on it. The mass of each atom is first calculated using Eq. (18). After that, the algorithm identifies *K* neighboring atoms with better fitness values according to Eq. (19).

18 $$\:{m}_{i}\left(t\right)=\frac{{e}^{-\frac{Fi{t}_{i}\left(t\right)-Fi{t}_{best}\left(t\right)}{Fi{t}_{worst}\left(t\right)-Fi{t}_{best}\left(t\right)}}}{\sum\:_{j=1}^{N}{e}^{-\frac{Fi{t}_{i}\left(t\right)-Fi{t}_{best}\left(t\right)}{Fi{t}_{worst}\left(t\right)-Fi{t}_{best}\left(t\right)}}}$$

where $\:{m}_{i}\left(t\right)$, *N*, $\:Fi{t}_{best}$, and $\:Fi{t}_{worst}$ are the mass of the *i-*th atom, number of atoms in the population, and fitness values of the best and worst atoms at the *i-*th iteration, respectively.

19 $$\:K\left(t\right)=N-\left(N-2\right)\times\:\sqrt{\frac{t}{T}}$$

where *T* is the maximum number of iterations. The interaction forces (attract or repel) and constraint force acting on the atom are calculated using Eqs. (20, 21), respectively.

20 $$\:{F}_{i}^{d}\left(t\right)=\sum\:_{j\in\:{K}_{best}}{rand}_{j}{F}_{ij}^{d}\left(t\right)$$

21 $$\:{G}_{i}^{d}\left(t\right)=\beta\:{e}^{\frac{-20t}{T}}\:\left({x}_{best}^{d}\left(t\right)-{x}_{i}^{d}\left(t\right)\right)$$

where $\:{K}_{best}$ is a subset of an atom population consisting of atoms with the best fitness value, $\:{rand}_{j}$, $\:\beta\:$, $\:{x}_{best}$, and $\:{x}_{i}$ denote a subset of an atom population consisting of atoms with the best fitness value, a random number in [0, 1], the multiplier weight, the position of the best atom, and the position of the *i-*th atom, respectively. The acceleration of the atom is determined by substituting the calculated forces and mass into Eq. (22), and the position of the atom is determined using Eqs. (23, 24), respectively. This process is repeated until the best fitness value is determined^61^.

22 $$\:{a}_{i}^{d}\left(t\right)=\frac{{F}_{i}^{d}\left(t\right)}{{m}_{i}^{d}\left(t\right)}+\frac{{G}_{i}^{d}\left(t\right)}{{m}_{i}^{d}\left(t\right)}$$

23 $$\:{v}_{i}^{d}\left(t+1\right)={rand}_{i}^{d}{v}_{i}^{d}\left(t\right)+{a}_{i}^{d}\left(t\right)$$

24 $$\:{x}_{i}^{d}\left(t+1\right)={x}_{i}^{d}\left(t\right)+{v}_{i}^{d}\left(t+1\right)$$

where $\:{v}_{i}$ and $\:{x}_{i}$ are the velocity and position of the *i-*th atom.

#### Parameters of the proposed algorithms

In this study, the hyperparameters of the CatBoost model were fine-tuned using three recent metaheuristic optimization algorithms: PPSO, DMO, and ASO, to enhance model performance. As a result, three hybrid frameworks combining CatBoost with each optimization technique were established. The integration of these optimizers into the CatBoost tuning workflow was achieved using the open-source Mealpy Python module^62^. CatBoost, a Python-based gradient boosting library, was employed for model construction. The internal settings of each optimizer listed in Table 2 based on recommendations by their respective developers^57^. The target hyperparameters optimized include the model’s depth (ranging from 1 to 10), learning rate (0.01 to 0.10), and number of iterations (100 to 1000), due to their known impact on prediction accuracy. The optimization process focused on minimizing the Root Mean Square Error (RMSE) by efficiently exploring these parameter ranges.

**Table 3:** Parameter values for the adopted models.

Hybrid model	Parameters
PPSO-CatBoost	Epoch = 500; pop_size = 30
DMO-CatBoost	Epoch = 500; pop_size = 30; n*_*babysitter = 3; peep = 2
ASO-CatBoost	Epoch = 500; pop_size = 30; alpha = 50; beta = 0.2

### Evaluation criteria

The evaluation of predictive models is fundamental to establishing their scientific credibility and practical applicability^63^. While training datasets assess a model’s capacity to fit the provided data, testing datasets are crucial for evaluating its ability to generalize to unseen data, mitigating overfitting and enhancing its applicability in real-world scenarios. This study adopts a dual evaluation approach, incorporating both visual and quantitative methods^64,65^. Visual tools, such as scatter plots, facilitate the analysis of the relationship between predicted and actual values, enabling the identification of patterns and anomalies that may not be evident through numerical measures. Quantitative evaluation, on the other hand, employs numerical metrics to objectively assess accuracy and reliability^66,67^, forming the basis for model comparison and selection^68^. Furthermore, uncertainty analysis is conducted to assess the robustness and reliability of the models^69^. Quantitative metrics play a pivotal role in providing objective and reproducible evaluations of model performance. Such measures are extensively used in research for rigorous model assessment and comparison^70^. In this study, six key metrics were selected to ensure a comprehensive evaluation: *R*^2^, RMSE, RMSRE, MAE, MAPE, and *U*_95_. These metrics, detailed in Table 4, offer valuable insights into the predictive accuracy, error magnitude, and reliability of the constructed models, facilitating the identification of the most effective predictive framework for the given task.

**Table 4:** Metrics for evaluating the performance of developed models.

Metric	Description	Equation
R^2^	Determination coefficient	$\:1-\frac{{\sum\:}_{i=1}^{n}{\left({y}_{i}-\widehat{{y}_{i}}\right)}^{2}}{{\sum\:}_{i=1}^{n}{\left({y}_{i\:}-\stackrel{-}{y}\right)}^{2}}$
RMSE	Root Mean Squared Error	$\:\sqrt{\frac{\sum\:_{i=1}^{\:n}{\left({y}_{i}-\widehat{{y}_{i}}\right)}^{2}}{n}\:}$
RMSRE	Root Mean Squared Relative Error	$\:\sqrt{\frac{1}{n}\sum\:_{i=1}^{\:n}{\left(\frac{{y}_{i}-\widehat{{y}_{i}}}{{y}_{i}}\right)}^{2}}$
MAE	Mean Absolute Error	$\:\frac{\sum\:_{i=1}^{\:n}\left|{y}_{i}-\widehat{{y}_{i}}\right|}{n}$
MARE	Mean Absolute Relative Error	$\:\sum_{i=1}^n\left|\frac{{y}_{i}-\widehat{{y}_{i}}}{{y}_{i}}\right|$
U_95_	Uncertainty Measure	$\:1.96\sqrt{{\text{Standard Deviation}}^{2}+{\text{RMSE}}^{2}}$

Where $\:\stackrel{-}{y}$ is the mean of actual values; $\:\stackrel{-}{\widehat{y}}\:$is the mean of predicted values; $\:{y}_{i}$ and $\:\widehat{{y}_{i}}\:$are actual and predicted *i*th values, respectively; *n* is the dataset number

### Feature importance analysis

Interpreting machine learning models is crucial for assessing their effectiveness^65^. SHAP is a popular method for feature sensitivity analysis, helping to understand how each input feature influences predictions. Derived from game theory, SHAP assigns an “importance value” to each feature by calculating its marginal contribution across different subsets of inputs. SHAP provides local interpretability, explaining individual predictions, which enhances transparency. Its consistency and additivity make it a reliable tool for analyzing feature importance in complex models.

## Results and discussion

### Statistical analyses

The multicollinearity analysis presented in Table 5 shows that the input variables are all statistically significant, as indicated by their strong T-statistics and low *p*-values. The VIF values for all variables are relatively low, with none exceeding the threshold that would suggest multicollinearity issues. For example, tributary area has a VIF of 1.171, superimposed load has a VIF of 1.053, formwork has a VIF of 1.709, and concrete has a VIF of 1.740. This indicates that there is minimal redundancy among the predictors, which reinforces the accuracy and reliability of the regression analysis. The findings highlight that the dataset utilized in this study is of excellent quality and well-suited for building strong predictive models.

**Table 5:** Results of multicollinearity analysis.

Input variables	Coefficients	Standard error	T-stat	*p*-value	Lower 95%	Upper 95%	*R* ^2^	VIF
Intercept	95.904	0.865	110.911	0.000	94.209	97.599	0.459	7.688
Tributary Area ($/m^2^)	23.216	1.183	19.633	0.000	20.899	25.534	0.459	1.171
Superimposed Load (kg/m^2^)	9.402	0.904	10.401	0.000	7.630	11.174	0.459	1.053
Formwork ($/m^2^)	4.304	1.688	2.550	0.011	0.996	7.613	0.459	1.709
Concrete ($/m^3^)	15.005	1.740	8.623	0.000	11.594	18.415	0.459	1.740

Table 6 presents the ANOVA results for the input variables, showing that they have statistically significant impacts on the output. For example, the F-statistics for tributary area (223.87), superimposed load (22.77), formwork (102.13), and concrete (52.15) are all substantially higher than the critical F-value of 3.8547, and their p-values are well below the standard significance threshold of 0.05. These findings indicate that variations in these factors are strongly associated with changes in the total construction cost, highlighting their importance as critical contributors to the model’s predictive performance.

**Table 6:** Results of ANOVA analysis analysis.

Variation source	SS	df	MS	F-statistic	*p*-value	F crit
ANOVA for Tributary Area ($/m^2^)						
Between groups	2.14 × 10^4^	1	2.14 × 10^4^	223.87	1.11 × 10^− 16^	3.8547
Within groups	6.71 × 10^4^	702	9.56 × 10^1^	–	–	-
Total	8.85 × 10^4^	703	–	–	–	–
ANOVA for Superimposed Load (kg/m^2^)						
Between groups	2.78 × 10^3^	1	2.78 × 10^3^	22.77	2.22 × 10^− 6^	3.8547
Within groups	8.57 × 10^4^	702	1.22 × 10^2^	-	–	–
Total	8.85 × 10^4^	703	–	–	–	–
ANOVA for Formwork ($/m^2^)						
Between groups	1.12 × 10^4^	1	1.12 × 10^4^	102.13	1.11 × 10^− 16^	3.8547
Within groups	7.73 × 10^4^	702	1.10 × 10^2^	–	–	–
Total	8.85 × 10^4^	703	-	–	–	–
ANOVA for Concrete ($/m^3^)						
Between groups	6.12 × 10^3^	1	6.12 × 10^3^	52.15	1.34 × 10^− 12^	3.8547
Within groups	8.24 × 10^4^	702	1.17 × 10^2^	-	–	–
Total	8.85 × 10^4^	703	–	–	–	–

Table 7 shows the Z-test results, which assess the statistical significance of the means of the input variables. The Z-scores for tributary area (55.65), superimposed load (45.57), formwork (252.06), and concrete (241.53) are exceptionally high, suggesting that their observed means are significantly different from a hypothesized mean of zero. These findings are further validated by the *p*-values, which are well below the critical thresholds for both one-tail (1.64) and two-tail (1.96) tests. This analysis strengthens the case for the influence of these input variables, confirming their reliability and significance within the dataset. In conclusion, the results from the multicollinearity, ANOVA, and Z-test analyses demonstrate that tributary area, superimposed load, formwork, and concrete are key variables with substantial impacts on the total construction cost. The high-quality dataset, coupled with the strong statistical support from these analyses, affirms the robustness of the predictive models developed.

**Table 7:** Z-test results for the input parameters.

Statistical parameter	Tributary Area ($/m^2^)	Superimposed Load (kg/m^2^)	Formwork ($/m^2^)	Concrete ($/m^3^)
Mean	45.91	475.23	54.50	90.50
Known Variance	479.08	76552.76	32.92	98.85
Observations	704.00	704.00	704.00	704.00
z-Score	55.65	45.57	252.06	241.53

### Sensitivity analysis

**Fig. 8:**
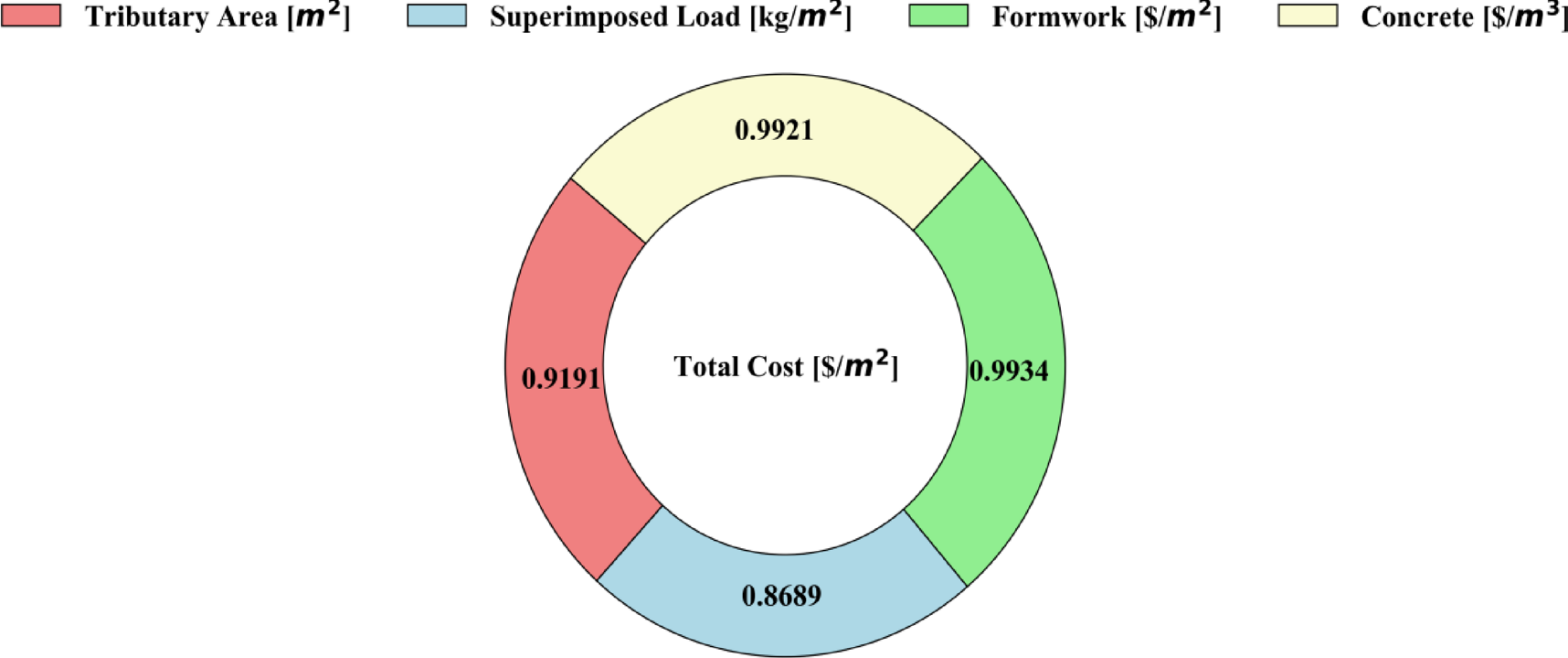
Sensitivity analysis of input variables.

Figure 8 shows the sensitivity analysis results for the total construction cost per square meter, depicted in a circular donut chart. This analysis reveals the relative significance of input variables such as tributary area, superimposed load, formwork cost, and concrete cost. Among these, the unit cost of concrete has the highest sensitivity value of 0.9934, indicating its major influence on the overall construction cost. This emphasizes the critical role of material selection and cost control in optimizing project expenses. The unit cost of formwork follows closely, with a sensitivity value of 0.9921, highlighting its substantial impact on cost variability. Proper design and efficient use of formwork materials are vital to minimizing construction expenses. The tributary area has a moderate sensitivity value of 0.9191, suggesting that while it plays a significant role in cost determination, its effect is less pronounced than material costs. The superimposed load demonstrates the lowest sensitivity value of 0.8689, indicating that while it influences cost, its impact is relatively minor compared to the other variables. Overall, the sensitivity analysis underscores the importance of managing material costs, particularly concrete and formwork, to effectively control total construction expenses. While the tributary area and superimposed load are less sensitive, they still warrant consideration to ensure a balanced and cost-effective design. This analysis provides valuable insights for prioritizing cost-related factors in predictive modeling and practical construction planning.

### Best hyperparameters

In Table 8, the best value for each hyperparameter of the hybrid models are presented. In order not to reduce the performance of the optimization algorithms, three hyperparameters of the CatBoost model, namely depth, learning_rate, and iterations, were optimized. Meanwhile, at most, three hyperparameters were optimized for the CatBoost model, because increasing the number of hyperparameters in the metaheuristic algorithm, such as PPSO, DMO, and ASO results, in a decrease in its performance. A limitation of this work is the limited number of hyperparameters available for optimization. Moreover, the hyperparameter values of the single CatBoost model, which has not undergone optimization of its parameters, are automatically set default values by the CatBoost module (depth = 3; learning rate = 0.1; iterations = 100).

**Table 8:** Best hyperparameter values obtained for the adopted models.

Hybrid model	Best hyperparameters
PPSO-CatBoost	Depth = 10; learning rate = 0.05; iterations = 368.40
DMO-CatBoost	Depth = 10; learning rate = 0.05; iterations = 500
ASO-CatBoost	Depth = 15; learning rate = 0.1; iterations = 821.09

### Predictive performance assessment

#### REC curves

**Fig. 9:**
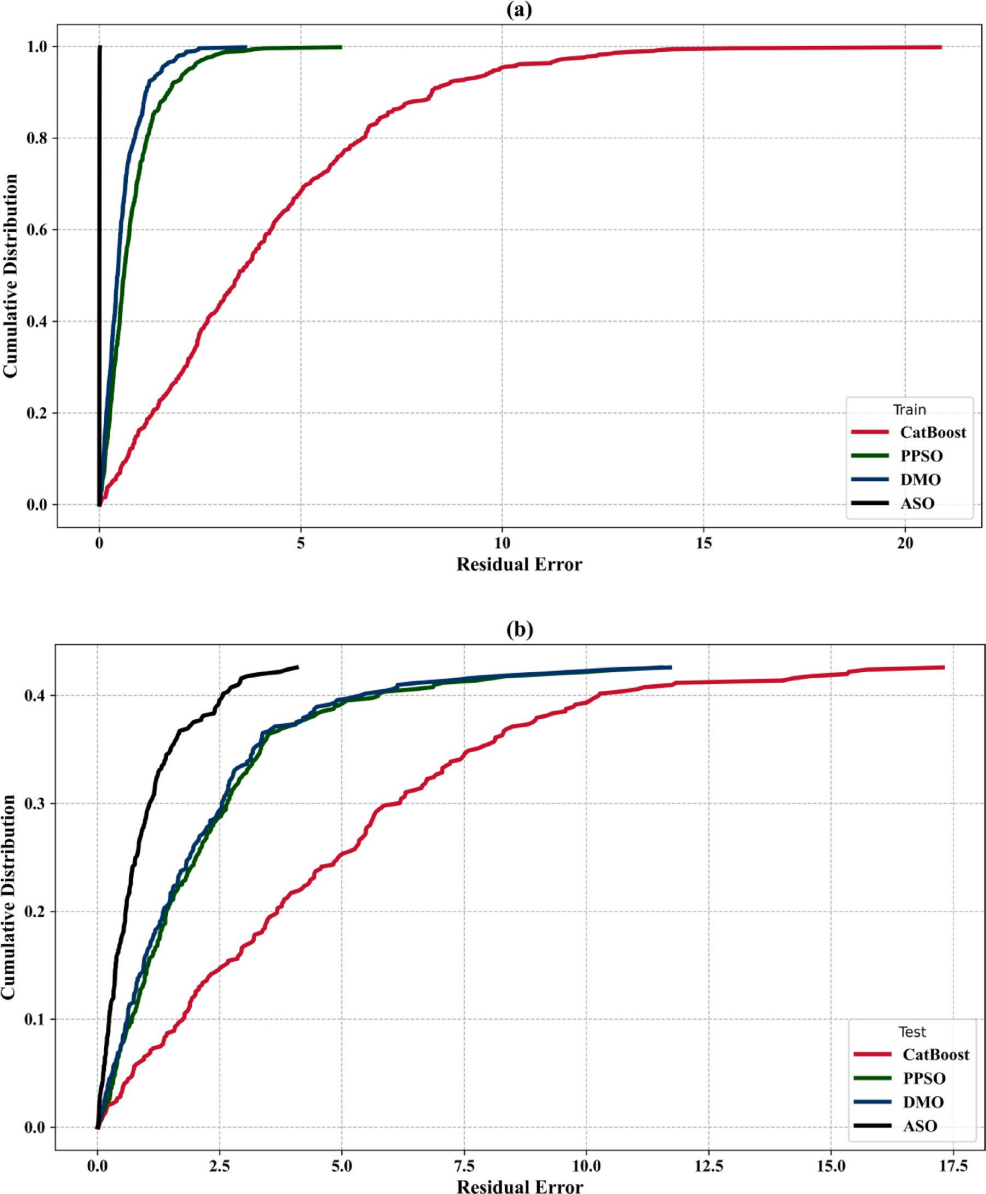
REC curves of the adopted models in the (**a**) training and (**b**) testing stages.

Figure 9 presents cumulative distribution plots of residual errors for the models used during both the training and test phases. In the training phase (Fig. 9a), CatBoost exhibits a wider range of residual errors, with a slower increase in the cumulative distribution compared to the other models. PPSO-CatBoost, DMO-CatBoost, and ASO-CatBoost show better performance, with steeper curves indicating fewer residual errors. In the test phase (Fig. 9b), CatBoost continues to demonstrate larger residual errors, while PPSO-CatBoost, DMO-CatBoost, and ASO-CatBoost maintain superior generalization, showing higher accuracy and fewer errors. Among the hybrid models, the PPSO-CatBoost model performs the best, consistently minimizing residual errors in both phases. Overall, the hybrid models outperform CatBoost in reducing errors across both phases.

#### Scatter plots

**Fig. 10:**
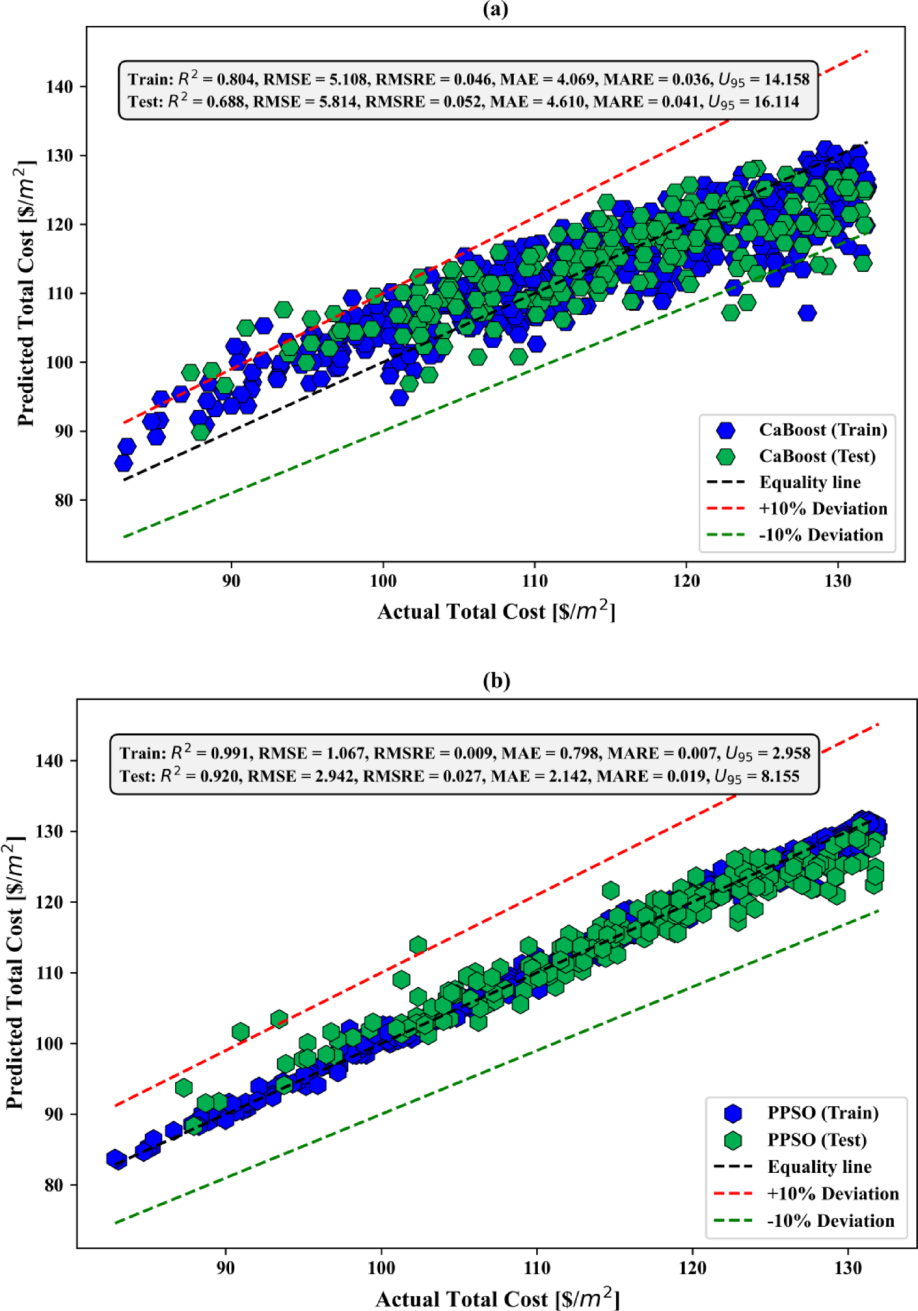

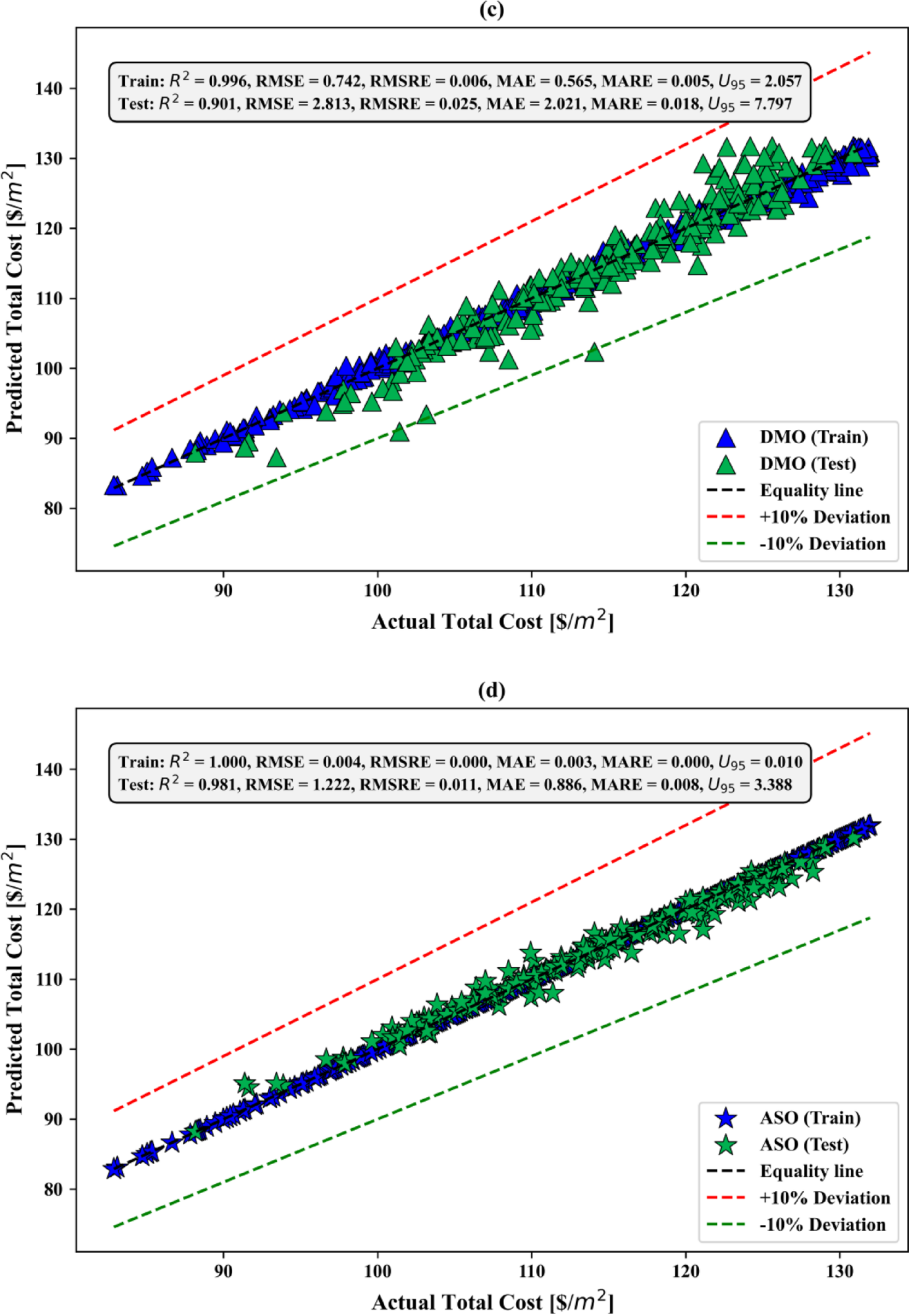
Performance scatter plots of the adopted models in the training and testing stages for (**a**) CatBoost, (**b**) PPSO-CatBoost, (**c**) DMO-CatBoost, and (**d**) ASO-CatBoost.

In Fig. 10a, the CatBoost model shows a broader spread of predicted values, with significant deviations from the equality line, particularly in the test phase. While the model performs reasonably well during training, its predictive accuracy deteriorates when applied to unseen data. Several test points fall outside the ± 10% deviation range, indicating that the model is prone to higher errors and struggles to maintain precision when generalizing to new data. This suggests a potential overfitting issue or a need for further tuning. In Fig. 10b, the PPSO-CatBoost model exhibits strong performance, with a close alignment between predicted and actual values in both the training and test phases. The model shows a good distribution of points along the equality line, with a moderate spread of residuals, especially during testing. Despite the slightly larger spread in the test phase, the majority of points remain within the ± 10% deviation range, suggesting reliable predictions and a relatively low margin of error.

In Fig. 10c, the DMO-CatBoost model performs effectively, with most of the predicted values closely following the equality line. The spread of residuals is relatively small, particularly in the training phase, and the test phase maintains good alignment, though with a slightly wider spread. The majority of the predictions fall within the ± 10% deviation lines, indicating a high degree of accuracy and reliability. The model generalizes well from training to testing, although there is a slight increase in error when tested on unseen data. In Fig. 10d, the ASO-CatBoost model demonstrates excellent predictive performance during both the training and test phases. The points are tightly clustered along the equality line, indicating a high level of agreement between the predicted and actual total cost values. The spread of residuals is minimal, with most data points falling within the ± 10% deviation lines. This suggests that the model generalizes well to unseen data and is highly accurate in both phases, showing minimal errors and maintaining consistency across the dataset. Overall, the ASO-CatBoost model stands out as the best performer, with minimal errors and the closest alignment to the equality line in both training and test phases, making it the most accurate and reliable model among those presented.

#### Violin boxplots

**Fig. 11:**
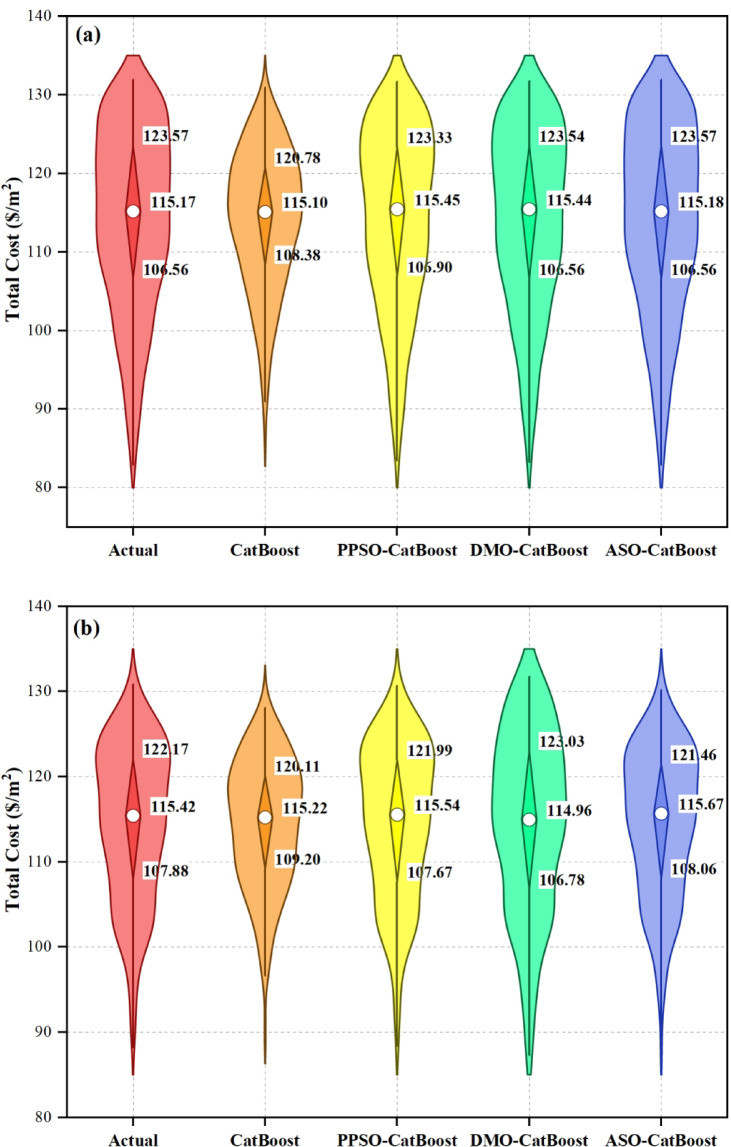
Violin boxplots illustrating the distribution of the output during (a) training and (b) testing stages.

Figure 11 presents violin plots comparing the distribution of predicted total costs for different models against the actual total costs in both training and test phases. In Fig. 11a, during training, CatBoost shows a wider spread and tends to overestimate the total cost compared to the actual value. In contrast, PPSO-CatBoost, DMO-CatBoost, and ASO-CatBoost demonstrate better accuracy, with ASO-CatBoost closely matching the actual cost and having the tightest distribution. In Fig. 11b, during testing, CatBoost again shows greater overestimation and variance. The hybrid models perform better, with ASO-CatBoost continuing to align closely with the actual total cost and maintaining a narrow distribution, indicating strong predictive accuracy and generalization. In both phases, ASO-CatBoost performs the best, offering the closest predictions and minimal spread.

### SHAP analysis

**Fig. 12:**
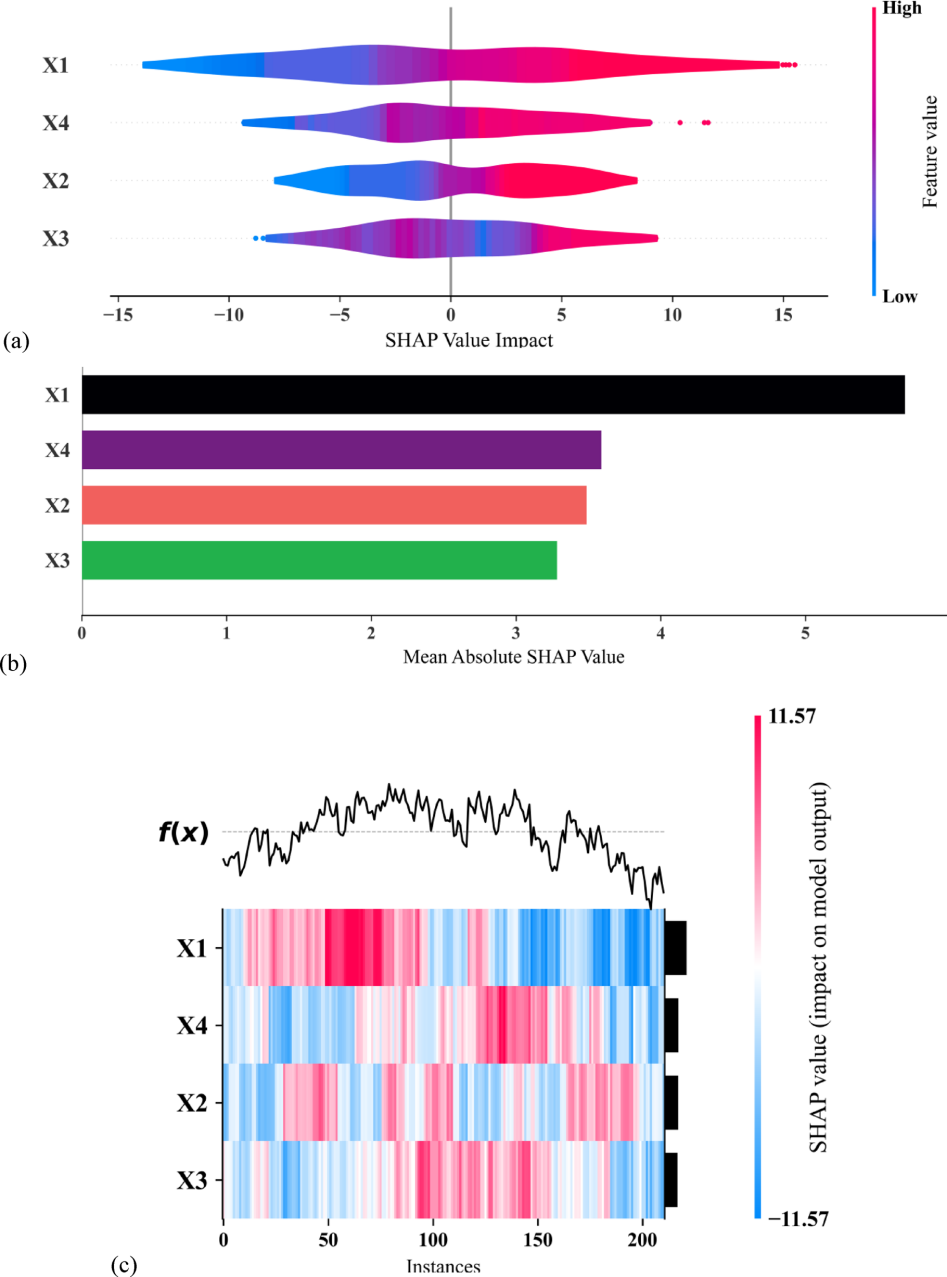
Feature importance analysis (**a**) summary violin plot, (**b**) summary bar plot, and (**c**) heatmap plot.

Figure 12 presents three SHAP plots, which help explain the contribution of each feature (X1, X2, X3, X4) to the model’s predictions. The features X1 to X4 represent the following: Tributary Area ($/m^2^) is X1, Superimposed Load (kg/m^2^) is X2, Formwork ($/m²) is X3, and Concrete ($/m^3^) is X4. Figure 12a shows the distribution of SHAP values for each feature and their corresponding impact on the model output. High SHAP values (in red) indicate a positive contribution, while low SHAP values (in blue) reflect a negative impact. X1 has the largest range of impact, with both high and low values influencing the model’s prediction, while X3 shows a smaller and more consistent impact. Figure 12b shows the mean absolute SHAP value for each feature, quantifying the overall contribution of each feature to the model. X1 is the most influential, followed by X4, X2, and X3. This plot indicates that X1 has the highest average contribution to the model’s predictions. Figure 12c provides a summary of the SHAP values across instances, illustrating how each feature contributes to the prediction across all instances. The red and blue colors represent positive and negative impacts, respectively. X1 and X4 exhibit strong and varying impacts across instances, while X2 and X3 have more moderate and consistent effects. Overall, the model shows that X1 is the most significant feature contributing to the model’s output.

### Interactive graphical user interface

**Fig. 13:**
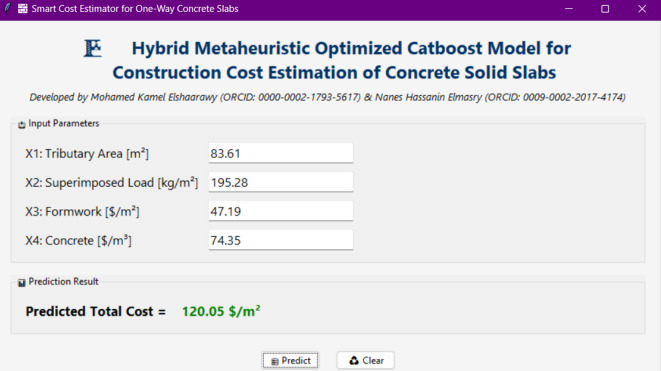
Screenshot of the developed GUI model for predicting construction cost of concrete slabs.

Figure 13 shows a user-friendly interactive GUI developed to bridge the gap between advanced machine learning models and practical applications. This Python-based web application, built using the Tkinter package, simplifies the deployment of the optimized model for engineers and designers^71^. The intuitive interface allows users to input model variables and instantly receive predicted outputs. By removing the complexities of database setup, model training, and validation, this tool makes advanced predictive capabilities more accessible. To encourage wider use and collaborative improvement, the GUI has been made available on GitHub, enabling its adaptation for various civil engineering applications^72–74^. Overall, this innovation provides an easy-to-use interface, facilitating the integration of machine learning models into real-world design tasks and advancing the field’s technological capabilities. The GUI can be accessed at https://github.com/mkamel24/concrete slabs.

The GUI presented is a prediction tool for estimating the total construction cost of concrete slabs using specified input parameters. It includes fields for four key inputs related to the design and material properties of the slab: the tributary area (X​1), the superimposed load (X2​), the unit cost of formwork (X3), and the unit cost of concrete (X4). After entering these parameters, the user can click the “Predict” button to calculate and display the predicted total cost ($/m^2^), which appears in the “Prediction Result” section. The GUI is designed for user convenience, featuring robust input validation mechanisms to ensure accuracy. For instance, non-numeric or invalid entries trigger error messages prompting the user to provide valid input values. This ensures that the prediction process is only initiated with realistic and accurate data.

In the example shown, the user inputs a tributary area of 83.61 m^2^, a superimposed load of 195.28 kg/m^2^, a formwork cost of 47.19 $/m^2^, and a concrete cost of 74.35 $/m^3^. Upon clicking the “Predict” button, the predicted total construction cost is displayed as 120.05 $/m^2^. This makes the tool highly efficient for engineers and construction professionals requiring quick and accurate cost estimations based on predefined slab specifications. If the user wishes to reset all input fields and clear the result, they can click the “Clear” button to start afresh. The tool provides a streamlined and effective solution for cost prediction tasks, aiding in the planning and optimization of construction projects. Future enhancements could include graphical visualizations or additional customization options to further enrich user experience and decision-making capabilities.

## Discussion

### Importance of statistical validation in model reliability

The statistical evaluation of the model inputs has demonstrated the significance of key variables such as tributary area, superimposed load, formwork, and concrete in determining construction costs. The absence of multicollinearity among the input variables, as evidenced by low VIF values, indicates that the predictors are independent and contribute uniquely to the model’s performance. Additionally, the ANOVA and Z-test results confirm that these variables significantly influence the total cost, ensuring the reliability and robustness of the dataset. This strong statistical foundation validates the predictive models developed in this study, making them highly dependable for practical applications in construction cost estimation. By ensuring the independence and relevance of input features, the model can deliver accurate predictions and provide actionable insights for construction planning.

### Performance enhancement through hybrid metaheuristic optimization

The integration of hybrid metaheuristic optimization techniques, such as PPSO, DMO, and ASO, with the CatBoost algorithm has significantly improved the accuracy of cost predictions. The results indicate that these hybrid models outperform the standalone CatBoost model, particularly in minimizing residual errors and maintaining generalization between training and testing phases. Among the hybrid approaches, ASO-CatBoost consistently demonstrates superior performance with minimal prediction errors and high alignment between predicted and actual values. This underscores the effectiveness of metaheuristic algorithms in optimizing hyperparameters, leading to improved model performance. The findings highlight the potential of hybrid optimization techniques as a valuable tool in enhancing the predictive accuracy of machine learning models in construction cost estimation.

### Practical implications for construction cost management

The sensitivity analysis and SHAP feature importance analysis provide valuable insights into the relative importance of input variables, with material costs such as concrete and formwork emerging as the most influential factors in total construction costs. These findings emphasize the critical role of material selection and cost control in construction project planning. The high sensitivity of concrete costs suggests that minor variations in this parameter can have a substantial impact on overall expenses, making it a key area for cost optimization. Similarly, the results demonstrate the importance of efficient design and usage of formwork to minimize cost variability. The analysis underscores the practical utility of the developed models in guiding resource allocation and decision-making to achieve cost-effective and balanced project outcomes.

The findings of this study align closely with the work of Chakraborty et al.^43^ which also identified material costs, particularly concrete and formwork, as dominant factors influencing construction expenses. Chakraborty et al.^43^ highlighted the critical importance of prioritizing these variables in cost management strategies to achieve substantial budgetary savings. The concurrence of results not only validates the reliability of the current study but also reinforces the broader applicability of these insights across different construction contexts. By focusing on high-impact variables, construction managers can make informed decisions to optimize project budgets and timelines while maintaining alignment with established research findings.

## Conclusions

This study introduces an advanced machine learning framework for predicting the construction cost of one-way solid concrete slabs by integrating the CatBoost model with three metaheuristic optimization algorithms: Phasor Particle Swarm Optimization (PPSO), Dwarf Mongoose Optimization (DMO), and Atom Search Optimization (ASO). These hybrid models were developed to fine-tune essential hyperparameters: depth, learning rate, and iterations, aiming to improve prediction accuracy and model robustness. Based on the results, the following key points can be concluded.

- The statistical evaluation confirmed that the input variables are all statistically significant and non-collinear. This ensured the reliability of the dataset and the foundation for developing robust models. Sensitivity analysis further highlighted that the unit costs of concrete and formwork had the greatest influence on construction cost, underscoring the importance of material selection and cost management in structural design.
- Performance comparisons demonstrated that all three hybrid models outperformed the standalone CatBoost model. Among them, ASO-CatBoost achieved the best results during the training stage, with an R^2^ of 0.9999 and RMSE of 0.004 $/m^2^, indicating an almost perfect fit. It also performed best in the testing phase, achieving an R^2^ of 0.981, RMSE of 1.222 $/m^2^, MAE of 0.886 $/m^2^, and MARE of 0.008. The model exhibited the lowest error dispersion and strongest generalization ability.
- DMO-CatBoost delivered consistent and reliable results, particularly in the test phase, with an R^2^ of 0.901, RMSE of 2.813 $/m^2^, MAE of 2.021 $/m^2^, and MARE of 0.018. PPSO-CatBoost also showed improvements over the baseline, recording a test R^2^ of 0.920, RMSE of 2.942 $/m^2^, MAE of 2.142 $/m^2^, and MARE of 0.019. In contrast, the standalone CatBoost model showed weaker performance, with a test R^2^ of 0.688, RMSE of 5.814 $/m^2^, MAE of 4.610 $/m^2^, and MARE of 0.041, indicating lower generalization and accuracy.
- SHAP analysis revealed that tributary area and concrete cost were the most influential predictors across all models, offering key insights for cost optimization and resource allocation in construction projects.
- To support practical implementation, a user-friendly graphical user interface (GUI) was developed using Python’s Tkinter library. This interface allows users to input project parameters and instantly receive cost predictions, eliminating the need for technical expertise in machine learning. The GUI enables real-time integration into engineering workflows and enhances accessibility for industry professionals.

## Study limitations and future work

A limitation of this study is the restricted number of hyperparameters optimized for the CatBoost model due to performance constraints of the metaheuristic algorithms. Optimizing more parameters could further enhance the model’s performance. Future investigations are encouraged to optimize a broader range of parameters and assess their impact on model performance. In addition, extending the study to incorporate other hybrid machine learning approaches and employing advanced feature selection techniques could lead to improved model robustness and reliability. Long-term validation using larger, diverse datasets and real-world construction data is recommended to assess the scalability and generalizability of the proposed models. Furthermore, although variables such as labor cost, reinforcement cost, concrete handling, and concrete type were not directly included, their combined effects are implicitly reflected in the unit costs of concrete and formwork. These values serve as composite indicators that encompass material, labor, and associated logistical factors. Future research may explore the inclusion of more detailed construction-related features to enhance model granularity and interpretability. Additionally, future improvements will focus on expanding GUI usability testing and refining the interface based on practical usage scenarios to enhance overall user experience and facilitate broader adoption in real-world design and construction cost management workflows.

## Data Availability

All data supporting the findings of this study are fully included within the manuscript.
